# Impact of Transcription Units rearrangement on the evolution of the regulatory network of gamma-proteobacteria

**DOI:** 10.1186/1471-2164-9-128

**Published:** 2008-03-17

**Authors:** Abel D González Pérez, Evelyn González González, Vladimir Espinosa Angarica, Ana Tereza R Vasconcelos, Julio Collado-Vides

**Affiliations:** 1 Centro Nacional de Bioinformática. Industria y San José, Capitolio Nacional, CP. 10200, Habana Vieja, Ciudad de la Habana, Cuba; 2 Departamento de Bioquímica y Biología Molecular y Celular, Facultad de Ciencias, Universidad de Zaragoza. Pedro Cerbuna 12, 50009 Zaragoza, España; 3 Instituto de Biocomputación y Física de Sistemas Complejos, Universidad de Zaragoza. Corona de Aragón 42 Edificio Cervantes, 50009 Zaragoza, España; 4 Laboratório Nacional de Computação Científica. Av. Getulio Vargas 333, Quitandinha, CEP 25651-075, Petropolis, Rio de Janeiro, Brasil; 5 Programa de Genómica Computacional, Centro de Ciencias Genómicas, Universidad Nacional Autónoma de México. Av. Universidad s/n., Colonia Chamilpa 62210, Cuernavaca, Morelos, México

## Abstract

**Background:**

In the past years, several studies begun to unravel the structure, dynamical properties, and evolution of transcriptional regulatory networks. However, even those comparative studies that focus on a group of closely related organisms are limited by the rather scarce knowledge on regulatory interactions outside a few model organisms, such as *E. coli *among the prokaryotes.

**Results:**

In this paper we used the information annotated in Tractor_DB (a database of regulatory networks in gamma-proteobacteria) to calculate a normalized Site Orthology Score (SOS) that quantifies the conservation of a regulatory link across thirty genomes of this subclass. Then we used this SOS to assess how regulatory connections have evolved in this group, and how the variation of basic regulatory connection is reflected on the structure of the chromosome. We found that individual regulatory interactions shift between different organisms, a process that may be described as rewiring the network. At this evolutionary scale (the gamma-proteobacteria subclass) this rewiring process may be an important source of variation of regulatory incoming interactions for individual networks. We also noticed that the regulatory links that form feed forward motifs are conserved in a better correlated manner than triads of random regulatory interactions or pairs of co-regulated genes. Furthermore, the rewiring process that takes place at the most basic level of the regulatory network may be linked to rearrangements of genetic material within bacterial chromosomes, which change the structure of Transcription Units and therefore the regulatory connections between Transcription Factors and structural genes.

**Conclusion:**

The rearrangements that occur in bacterial chromosomes-mostly inversion or horizontal gene transfer events – are important sources of variation of gene regulation at this evolutionary scale.

## Background

The transcriptional regulatory network (TRN) defines the cellular response to environmental stimuli at the step of transcription initiation. It may be analyzed at four different levels of increasing complexity: individual regulatory interactions, network motifs, functional modules, and the entire network that may be represented as a directed graph [1]. Several studies in the past years have begun to unravel the structure, dynamical properties, and evolution of the network at these four levels of organization [2-7] using mainly as a starting point the information of the regulatory networks of *E. coli *and *S. cerevisiae*.

Studies that focus on the evolution of the regulatory network, even within a group of closely related organisms are limited by the rather scarce knowledge on regulatory interactions outside a few model organisms. Among prokaryotes, *E. coli *K12 [8] and *B. subtilis *[9] have the best known and better annotated networks. Several approaches have been developed to overcome the lack of comparative information on regulatory interactions that is necessary in works aimed at studying the evolution of regulatory networks. Most of these approaches combine the use of sequence profiles of the sites recognized by a given Transcription Factor (TF) in a model organism (for instance *E. coli*) with orthology information between the genes of closely related organisms (based on the model organism) to identify putative orthologous regulatory interactions in organisms other than the model [5,10-18].

The problem is more challenging when one intends to extrapolate the information of known TRNs to phylogenetically distant organisms. Two recent studies [7,19] took the TRNs of *E. coli *and *B. subtilis *and assessed the conservation of their transcription factors (TFs) and regulated genes across a broad array of sequenced genomes. Both works showed that the set of regulatory genes – even global transcription factors – clearly varies from one group of organisms to another. On the other hand, several studies have shown that, within a group of closely related organisms, the conservation of a TF and a subset of the structural genes it regulates are not sufficient to assume that regulatory links between them are conserved between such two organisms [10-18].

Therefore, the question whether the interchange of regulatory links between TFs and structural genes could be a significant mechanism to "rewire" the regulatory network between closely related organisms remains open. This rewiring has been compared to changing the "software" of the transcriptional regulation machinery [20], as opposed to changing the "hardware". The latter would mean changing the set of TFs or regulated genes – a process observed by Babu *et al*. [7] and Lozada-Chavez *et al*. [16] – between phylogenetically distant organisms.

In this paper we use the information stored in Tractor_DB to compute a normalized Site Orthology Score (SOS) that quantifies the conservation of a regulatory interaction across thirty genomes currently included in the database. We then use this SOS to assess the evolution of individual regulatory interactions and to find out whether the rearrangement of these connections affects the conservation of network motifs. We found that, within the evolutionary scale of the members of the gamma-proteobacteria subclass, the regulatory links that form feed forward motifs are more correlatedly conserved than triads of random regulatory interactions or pairs of co-regulated genes.

Moreover, we used the information stored in Tractor_DB together with the information on the organization of gamma-proteobacterial genomes in Transcription Units (TUs) [21] to assess whether there is a correlation between the conservation of a regulatory site and the structure of the TU whose expression it controls. We found, in agreement with a previous result obtained for seven organisms [5], that regulatory sites with higher SOS are more likely to occur upstream TUs with more conserved structures, suggesting that a correlation between the conservation of a regulatory site and the conservation of the structure of the TU it regulates does exist at this evolutionary scale. With these results and those obtained by Belda *et al*. [22] regarding genome rearrangements in the gamma-proteobacteria, we state the hypothesis that genome rearrangement events, such as inversion and horizontal gene transfer events, that have occurred during the evolution of this group may have produced TU rearrangements that caused the rewiring that is observed within the network. These changes in the regulation of individual structural genes would be fixed depending on the lifestyle of each organism [7,13]. Therefore, while gene duplication and deletion events may be responsible for changing the TRN "hardware" over large evolutionary scales, operon rearrangements may play a major role in reprogramming at the "software" level within groups of closely related organisms.

## Results

As previously stated, given the scarcity of experimentally verified regulatory sites in gamma-proteobacterial genomes, our study is based entirely on computational predictions obtained from Tractor_DB. In order to validate the robustness of these predictions we a) calculated the sensitivity and specificity of the computational approaches taken to produce them; b) compared the *E. coli *experimentally verified TRN, and the *E. coli *and *S. typhimurium *reconstructed TRNs with data from gene expression experiments; c) assessed the circularity of the populations of predicted sites, and; d) tested the possibility of a correlation between the SOS of *E. coli *regulatory sites and the scores of their orthologs. (See Discussion for details.) The starting dataset comprises all putative regulatory interactions found within the genomes of the thirty gamma-proteobacteria included in the database. Table 1 presents a summary of this data, namely the number of putative regulatory interactions annotated in Tractor_DB, as well as the number of Regulons and TUs for which regulatory information is available, for each organism. Each interaction connects a TF to a structural gene; therefore, a regulatory site (or several sites, as mentioned above) identified upstream a TU defines regulatory links that connect the TF that binds to the site with each gene of the TU (datasets corresponding to the regulatory networks of the organisms used in this study may be downloaded from Tractor_DB). The number of regulatory interactions identified in the networks of each organism ranges from 2,118 in *E. coli *K12 to 6 in *X. axonopodis *and *M. capsulatus*. Table 1 also presents the Accession Numbers to the genomic sequences of all organisms included in this study, as well as abbreviated names for each organism that are used elsewhere in the paper (for further details concerning the computational genomics approaches used to produce these data, please refer to Additional file 1).

**Table 1:** Summary of the starting data set, with organisms ordered by number of identified interactions

**Organism name**	**Abbreviated Name**	**Accession Number**	**Interactions**	**Regulons**	**TUs**
*Escherichia coli *K12	Eco	[GenBank: NC_000913]	2118	87	938
*Salmonella typhimurium *LT2	Stm	[GenBank: NC_003197]	1901	69	832
*Salmonella typhi *CT18	Sty	[GenBank: NC_003198]	1871	68	812
*Shigella flexneri *2a 301	Sfle	[GenBank: NC_004337]	1465	62	718
*Escherichia coli *O157H7	Ecoo	[GenBank: NC_002655]	862	46	411
*Yersinia pestis *KIM	Ypes	[GenBank: NC_004088]	740	28	405
*Shigella flexneri *2a 2457T	Sfl2	[GenBank: NC_004741]	725	47	367
*Shewanella oneidensis *MR-1	Sone	[GenBank: NC_004347]	573	20	345
*Vibrio cholerae *N16961	Vcho	[GenBank: NC_002505, GenBank: NC_002506]	506	22	267
*Haemophilus influenzae *Rd KW20	Hinf	[GenBank: NC_000907]	350	19	193
*Vibrio parahaemolyticus *RIMD 2210633	Vpar	[GenBank: NC_004603, GenBank: NC_004605]	305	29	182
*Yersinia pseudotuberculosis *IP32953	Ypse	[GenBank: NC_006155]	297	26	185
*Erwinia carotovora *SCRI1043	Ecar	[GenBank: NC_004547]	295	7	18
*Yersinia pestis Mediaevails *91001	Ypem	[GenBank: NC_005810]	292	25	183
*Photorhabdus luminescens *TT01	Plum	[GenBank: NC_005126]	256	23	136
*Vibrio vulnificus *CMCP6	Vvul	[GenBank: NC_004459, GenBank: NC_004460]	256	26	157
*Photobacterium profundum*	Ppro	[GenBank: NC_006370, GenBank: NC_006371]	156	21	104
*Haemophilus ducreyi *35000HP	Hduc	[GenBank: NC_002940]	106	12	60
*Acinetobacter sp *ADP1	Aadp	[GenBank: NC_005966]	71	9	41
*Pseudomonas aeruginosa *PA01	Paer	[GenBank: NC_002516]	42	11	24
*Pseudomonas putida *KT2440	Pput	[GenBank: NC_002947]	36	9	17
*Pseudomonas syringae *DC3000	Psyr	[GenBank: NC_004578]	34	11	18
*Buchnera aphidicola *Bp	Baph	[GenBank: NC_004545]	31	8	22
*Legionella pneumophila *Paris	Lpnp	[GenBank: NC_006368]	27	8	11
*Legionella pneumophila *Philadelphia	Lpnh	[GenBank: NC_002942]	20	8	8
*Xylella fastidiosa *9a5c	Xfas	[GenBank: NC_002488]	18	6	9
*Legionella pneumophila *Lens	Lpnl	[GenBank: NC_006369]	16	6	9
*Xanthomonas campestris *ATC 33913	Xcam	[GenBank: NC_003902]	8	5	6
*Methylococcus capsulatus *Bath	Mcap	[GenBank: NC_002977]	6	3	3
*Xanthomonas axonopodis *306	Xaxo	[GenBank: NC_003919]	6	3	4

### Variability of the gene content of orthologous regulons

Previous studies done in different groups of gamma-proteobacteria have shown that the gene content of a few individual regulons (such as CRP, FNR, Fur, Zur, LexA, and KdpE) changes from one organism to another [10-18]. This phenomenon does not depend on the substitution of the set of TFs and structural genes that takes place between more distant organisms, as observed by Babu *et al*. [7] and Lozada-Chavez *et al*. [19]. Nevertheless, both processes have been explained in terms of the diverse regulatory needs that have arisen in different organisms as they adapt to different lifestyles, which are defined by Babu *et al*. [7] as a combination of four properties: oxygen requirement, optimal growth temperature, environmental conditions, and whether the organism is pathogenic or not.

**Figure 1:**
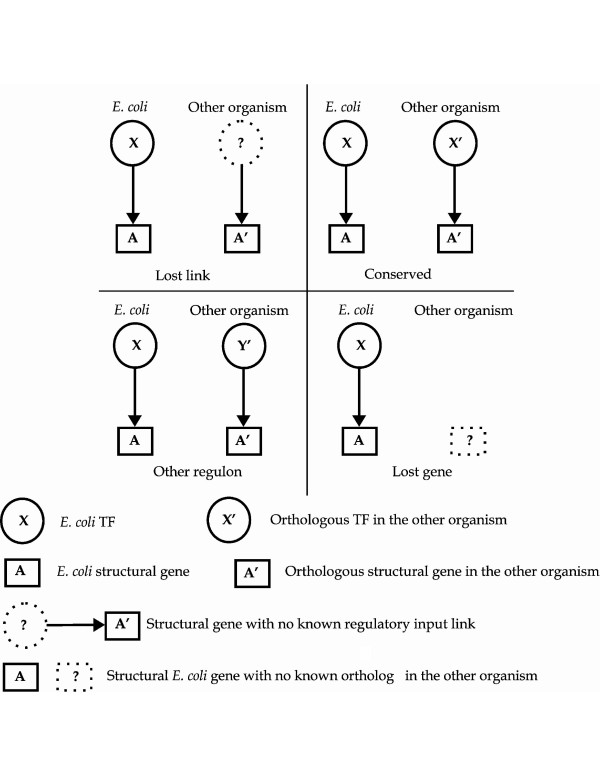
**Conservation of orthologous regulatory interactions**. Each orthologous regulatory interaction is classified according to its conservation with respect to *E. coli *regulatory interactions (see detailed explanation in the text).

To determine whether this variation in gene content is a general property of the regulatory network, we classified all orthologous regulatory interactions in our starting dataset (or their absence) into four groups depending on their conservation with respect to *E. coli *regulatory interactions (see Methods and Figure 1). For that, interactions were labeled as **conserved**, **lost gene**, **other regulon **or **lost link under the assumption that **that the *E. coli *TF has an ortholog in the organism under analysis and all, except **lost gene, **are referred to orthologs of the *E. coli *gene. Therefore *an E. coli *regulatory interaction (or link) for which both an orthologous TF and an orthologous gene exist in another organism may be **conserved **(if an interaction links the orthologous TF and the orthologous gene); alternatively it may have changed to **other regulon **(if the orthologous gene is regulated by a different TF), or it may be **lost **(if no incoming interaction has been identified for the orthologous gene). This classification scheme, although based on *E. coli *(which is the original source for the computational predictions), intends to illustrate a process of disappearance of interactions and appearance of new regulatory links occurred within the evolutionary divergence of the different gamma-proteobacteria from their last common ancestor. See Additional file 2 for further discussion and illustration.

**Figure 2:**
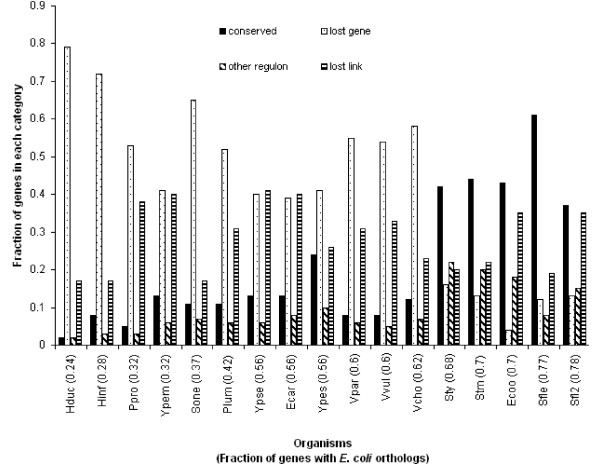
**Distribution of orthologous regulatory interactions in 17 organisms in the four conservation categories**. The organisms in the abscissa are ordered according to the fraction of their genes that have orthologs in *E. coli*. From left to right, solid black columns: **conserved **category; dotted columns: **lost gene **category; diagonal stripes: **other regulon **category; horizontal stripes: **lost link **category.

The fraction of orthologous interactions labeled as **other regulon **or **lost link **in other organisms ranges from approximately 18% (Hduc) to 55% (Ecoo). Figure 2 presents the distribution of orthologous interactions in the four previously described categories for 17 organisms (those with more than 100 regulatory interactions in the starting dataset), sorted by increasing fraction of genes with orthologs in *E. coli*. Regulatory interactions classified as **other regulon **indicate that a process of disappearance of interactions and appearance of new regulatory links – that may be described as a rewiring of the network – takes place at the most basic level of the network: the individual regulatory connections. A number of **lost links **may in fact be false negatives of the computational strategies that we used to rebuild the regulatory networks of the 30 organisms included in Tractor_DB. (Several effects such as the divergence of the sequence motif recognized by orthologous TFs may be the cause of these *E. coli *sites without orthologs.) However, given the results of the calculation of the rates of false negatives in the *E. coli *genome in the starting dataset (see Additional file 3) we are confident that many interactions classified as **lost links **indeed correspond to changes of transcriptional regulation between orthologous genes.

The SOS (see Methods) of *E. coli *regulatory connections is another way to look at their conservation (or variability) across the gamma-proteobacteria included in Tractor_DB. Additional file 4 presents significant values (mean, standard deviation and maximum) of the distribution of SOS values obtained for the 85 regulons included in Tractor_DB. Considering the network as a whole, the five regulons that present the highest mean SOS values (and therefore possess on average better conserved interactions) are PdhR (0.88), MarA (0.81), GlcC (0.7), TreR (0.68), and XylR (0.56). The distribution of SOS values in the entire network has a mean of 0.21 and a standard deviation of 0.07.

Table 2 exemplifies the aforementioned rewiring process for three regulons: FruR, NtrC and PurR (for each regulon only connections with at least the mean SOS for the regulon minus one standard deviation are shown). SOS values of the *E. coli *regulatory interactions range between 0.1 and 0.69 in the FruR regulon, and between 0.1 and 0.71 in the NtrC and the PurR regulons. Since the SOS is the quotient of two weighed summations (see Methods), low SOS values may be interpreted as regulatory interactions that are preferentially conserved in organisms that are closer to *E. coli*, and lost in more distant ones, while higher SOS values correspond to interactions that are conserved both in close and in distant organisms. For instance, the glutamine synthetase gene (*glnA*) is regulated by NtrC in *E. coli*. Orthologs of both the TF and the gene exist in other 29 genomes analyzed. We identified orthologous regulatory connections in 19 organisms only but because this list includes both close and distant genomes, the SOS produced is rather high. Actually, only 2% of all *E. coli *regulatory interactions have SOS values higher than 0.7. In the other extreme, the *codB *and *codA *genes form a Transcription Unit with orthologs in 20 of the genomes included in Tractor_DB, where their regulator (PurR) is also conserved. However, the regulatory link between them is only conserved in *S. typhi *and *S. typhimurium*, producing an SOS value of only 0.1, which is below the mean value of the network.

**Table 2:** Genes of three E. coli regulons sampled using the mean SOS minus one standard deviation (Cutoff values are shown in parenthesis in the leftmost column.)

**Regulon**	**Gene**	**SOS**	**Product**
**FruR (0.1)**	*purB*	0.44	adenylosuccinate lyase
	*fruA*	0.69	PTS family enzyme IIB'BC, fructose-specific
	*fruB*	0.69	PTS family enzyme IIA (N-terminal); FPr (C-terminal), fructose-specific
	*fruK*	0.69	fructose-1-phosphate kinase
	*icda*	0.54	e14 prophage; isocitrate dehydrogenase, specific for NADP+
	*aceB*	0.12	malate synthase A
	*aceA*	0.12	isocitrate lyase
	*setB*	0.23	lactose/glucose:proton efflux pump
	*glk*	0.15	glucokinase
	*epd*	0.46	D-erythrose 4-phosphate dehydrogenase
	*yibO*	0.14	phosphoglycerate mutase III
	*aroP*	0.17	aromatic amino acid transport protein
	*tpiA*	0.46	triosephosphate isomerase
	*pfkA*	0.46	6-phosphofructokinase I

**NtrC (0.11)**	*glnG*	0.55	NtrC regulator
	*glnL*	0.55	histidine protein kinase sensor for NtrC regulator
	*glnA*	0.71	glutamine synthetase
	*glnH*	0.11	high-affinity glutamine transport protein
	*glnK*	0.31	nitrogen regulatory protein P-II 2
	*amtB*	0.31	probable ammonium transporter

**PurR (0.09)**	*codB*	0.1	cytosine permease
	*codA*	0.1	cytosine deaminase
	*tsx*	0.12	nucleoside channel
	*purK*	0.37	phosphoribosylaminoimidazole carboxylase (CO_2 _fixating subunit)
	*purE*	0.37	phosphoribosylaminoimidazole carboxylase (catalytic subunit)
	*prsA*	0.1	phosphoribosylpyrophosphate synthetase
	*purR*	0.1	repressor for purine nucleotide synthesis
	*purT*	0.71	phosphoribosylglycinamide formyltransferase 2
	*purF*	0.48	amidophosphoribosyltransferase
	*cvpa*	0.48	membrane protein required for colicin V production
	*upp*	0.35	uracil phosphoribosyltransferase
	*purC*	0.18	phosphoribosylaminoimidazole-succinocarboxamide synthetase
	*purM*	0.38	phosphoribosylaminoimidazole synthetase
	*purN*	0.38	phosphoribosylglycinamide formyltransferase 1
	*glyA*	0.22	serine hydroxymethyltransferase
	*purL*	0.42	phosphoribosylformyl-glycineamide synthetase
	*yfhD*	0.42	putative periplasmic binding protein of transport system
	*gltS*	0.15	glutamate transport
	*yicE*	0.33	putative purine/xanthine transport protein
	*purH*	0.47	IMP cyclohydrolase (N-terminal); phosphoribosylaminoimidazolecarboxamide formyltransferase (C-terminal)
	*purD*	0.47	phosphoribosylglycinamide synthetase

**Figure 3:**
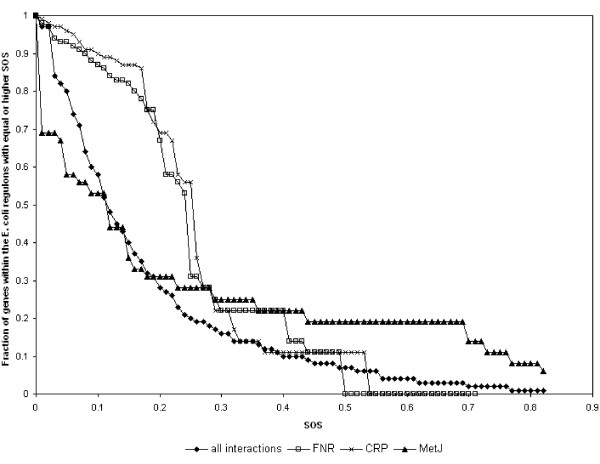
**Decay of the fraction of the E. coli regulatory interactions with increasing values of SOS**. For each SOS value represented in the graph, regulatory interactions with lower or equal SOS are sampled and counted, and the fraction they represent with respect to all interactions is computed. All interactions: samples are taken from the entire network; FNR, CRP, MetJ: samples are taken only from these regulons.

We also analyzed the distribution of the SOS values of regulatory links within the network by computing what fraction of regulatory interactions have SOS equal to or greater than an increasing cutoff value. This means sampling the regulatory interactions with SOS higher than a given increasing value. As Figure 3 shows (for all the interactions of the *E. coli *network and those of the CRP, FNR, and MetJ regulons separately) the fraction of regulatory interactions sampled decreases rapidly with the increase of the value of SOS used as cutoff for sampling. For instance, for the FNR regulon, the fraction drops to 0 at SOS 0.5, which means that no FNR outgoing interactions have associated SOS values higher than 0.5. In the entire *E. coli *network, only 8 regulatory interactions have SOS associated values higher than 0.8.

### Evolution of small network circuits

One question that arises concerning this rewiring of the network is whether local medium-level structures are affected by the appearance and disappearance of regulatory interactions. Regulatory interactions in the TRN form recurrent circuits that involve one, three or four nodes, and which are often referred to as network motifs [23,24]. Network motifs are known to be involved in local dynamic properties of the network, [4,25] and therefore, it would be interesting to determine whether *E. coli *motifs are conserved in other organisms to a higher extent than expected from the observed pattern of shifting of individual interactions.

To address this question, we extracted all the auto-regulatory loops (ALs: where a TF regulates the transcription of its own gene and the feed forward (FF: where two TFs, X and Y regulate the expression of gene A) motifs in the *E. coli *TRN reconstructed in Tractor_DB. For both types of motifs we used a metrics that is more elaborated than simply counting the number of conserved motifs in other organisms. We relied on the SOS of individual interactions to evaluate the conservation of motifs, because the SOS takes different values depending on which organisms bear orthologs to an *E. coli *regulatory site.

In the case of ALs, we calculated the mean of the associated SOS values of the interactions between the TF and its coding gene, and compared it with the average mean that resulted from randomly taking 1,000 samples composed of the same number of interactions than the set of ALs. As shown in Table 3, the mean SOS associated with regulatory interactions involved in ALs is 0.36, whereas the mean SOS of all regulatory interactions in the network is 0.21. We found that regulatory interactions that form ALs are on average better conserved than groups of interactions of the same size sampled randomly from the network (p-value 3.02*10^-12^). This result implies that natural selection acts at the level of motifs favoring the preservation of regulatory interactions that form auto-regulatory circuits – through which TFs control the transcription of their own genes – in this group of organisms.

**Table 3:** Conservation of regulatory links that form network motifs across the 30 genomes in the dataset.

**Mean SOS of regulatory interactions involved in AL motifs**				
**AL motifs in the *E. coli *network**	**Mean SOS**	**Mean SOS (whole network)**	**Z score (1000 random samples)**	**p-value**

28	0.36	0.21	7.89	3.02*10^-12^

**Correlations between the SOSs of pairs of interactions involved in FF motifs**				

**FF motifs in the *E. coli *network**				111

**Pair of interactions**	**Correlation**	**Z score (1000 random samples)**		**p-value**

TF_1_-gene/TF_2_-gene	0.78	5.15		2.6*10^-7^
TF_1_-TF_2_/TF_1_-gene	0.74	5.51		3.6*10^-8^
TF_1_-TF_2_/TF_2_-gene	0.64	5.07		3.98*10^-7^
Pairs of co-regulated genes	0.52	3.23		4.5*10^-4^

With regard to FF motifs, we assessed whether the regulatory interactions that form them are correlatedly conserved (see Methods). Again, since the SOS of a given *E. coli *interaction depends on which organisms have orthologous interactions, it may be seen as a phylogenetic profile measurement that takes similar values for two interactions when they are conserved in similar sets of organisms. Therefore, a positive correlation between the SOS values of two interactions implies that they appear in a similar set of organisms and are absent in a similar set. Table 3 shows that these three correlations are positive, and range from 0.64 to 0.78 (with p-values between 3.8*10^-8 ^and 3.98*10^-7^). These results mean that the profile of the conservation of two regulatory interactions that make part of an *E. coli *FF motif – across the genomes of the other 29 organisms in the dataset – tend to be more similar than the patterns of conservation of any two interactions sampled randomly from the network, which implies that the structure of *E. coli *FF motifs tends to be altogether conserved or lost in the same arrays of organisms, as defined by similar SOS. In other words, the regulatory links that form feed forward motifs [26,27] are conserved in a better correlated manner than triads of random regulatory interactions.

In order to compare the correlation of the SOS of interactions involved in FFs to other non-random structures within the network, we calculated the correlation coefficient of the SOS of 4089 pairs of *E. coli *co-regulated genes. Table 3 shows that, although statistically significant (p-value 4.5*10^-4^), the pairs of co-regulated genes as a rule, tend to be less correlatedly conserved than pairs of interactions involved in FF motifs.

### Conservation of the connectivity distribution

The connectivity distribution (i.e., the distribution of regulatory inputs received by TUs, and the distribution of regulatory outputs of TFs) in the transcriptional regulatory network may be approximated by a power law function with negative exponent [28-30]. We constructed the connectivity distribution for each organism with more than 250 regulatory interactions in the dataset to determine how the rewiring process that takes place at the most basic level of the network affects its global properties.

**Figure 4:**
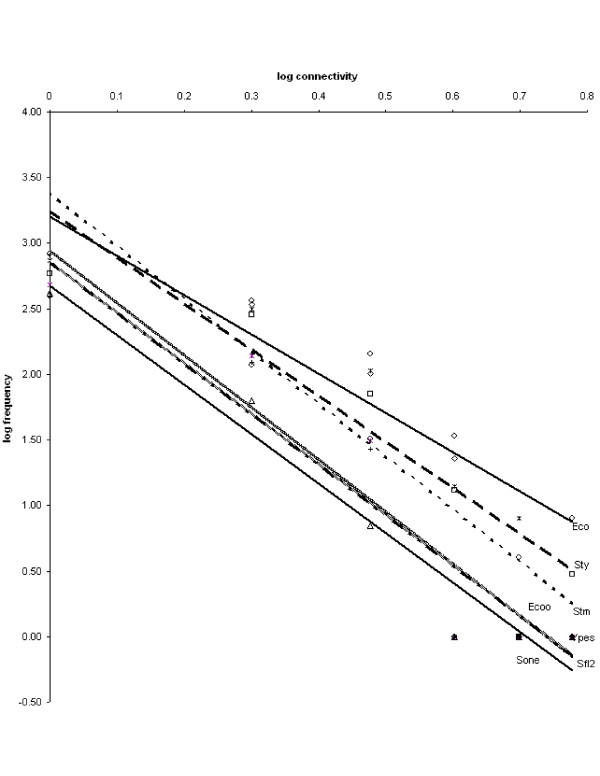
**Distribution of connectivity in different TRNs**. Connectivity distribution in the regulatory networks of Eco, Sty, Stm, Ecoo, Ypes, Sfl2 and Sone.

Figure 4 presents the incoming connectivity distribution – plotted as the logarithm of the frequency with which structural genes with a given number of incoming regulatory interactions appear in the network, against the logarithm of the number of incoming interactions – of the regulatory networks with more than 550 interactions (Eco, Sty, Stm, Ecoo, Ypes, Sfl2 and Sone). The slopes of the adjusting lines are very similar for all networks, ranging from -4 (Eco) to -2.98 (Stm); r^2 ^coefficients range from 0.85 to 0.88. These trend lines should not be taken as a golden standard because only a fraction of structural genes were included in the calculation. We use them to merely illustrate the similarity of their slopes between different organisms.

This result (which we had previously obtained for a smaller number of networks with fewer interactions [31]) suggests that despite the rewiring process that takes place at the most basic level of the network, the global properties of the graph, such as the connectivity distribution, remain unaffected within this group of organisms.

### The conservation of a regulatory site and the conservation of the regulated TU

Changes in gene regulation, that we detect as regulatory links that fall within the categories **other regulon **and **lost link **(see above) may have occurred by two main different events: either the gene was "relocated" to a different TU in one of the organisms resulting from a speciation event, or the orthologous regulatory regions diverged to an extent that they no longer bear sites recognized by the orthologous TFs (represented by orthologous weight matrices in the approach used to identify regulatory sites in Tractor_DB). In an attempt to assess the relative contribution of these two processes to changes in gene regulation, we computed the fraction of TUs in each organism whose structure is identical (see Methods) to their orthologous TUs in *E. coli *and bear genes with regulatory links either unknown (**lost link**) or that have switched to a different regulator (**other regulon**). We found that a fraction of the genes in these two categories ranging from 46% (*P. aeruginosa*) to 77% (*E. coli *O157H7) appear in TUs that are identical to their *E. coli *orthologs and therefore the change in regulation in their cases could only be explained by divergence of the orthologous regulatory regions.

This result suggests that TU rearrangements have caused the regulatory shift of a number of those genes whose regulation has changed with respect to their *E. coli *orthologs (from 23 to 54% depending on the organism). To test this hypothesis, we searched for any discernible relationship between the degree to which a regulatory site is conserved across the genomes of the organisms included in Tractor_DB, and the conservation of the structure of the regulated TUs. To assess this relationship, we classified the structure of each TU in one of four categories, according to its similarity to the structure of its *E. coli *orthologous TU(s). Sorted in order of decreasing conservation, the categories are Identical, Similar, Destroyed, and Lost (see Methods, and Itoh *et al*. [32] for details on these categories). TUs were also classified in four groups according to the SOS of the regulatory sites upstream them (clearly, only TUs with putative regulatory sites were included in this part of the study). The four groups were formed by the TUs with regulatory sites with SOS between 0 and 0.13, between 0.14 and 0.36, between 0.37 and 0.59, and between 0.6 and 1. We chose these extreme values for the intervals instead of evenly dividing the data in quarters, because the last quarter interval (0.75–1) would be highly under-represented due to the relative scarcity of interactions with SOS values higher than 0.75 [5].

**Figure 5:**
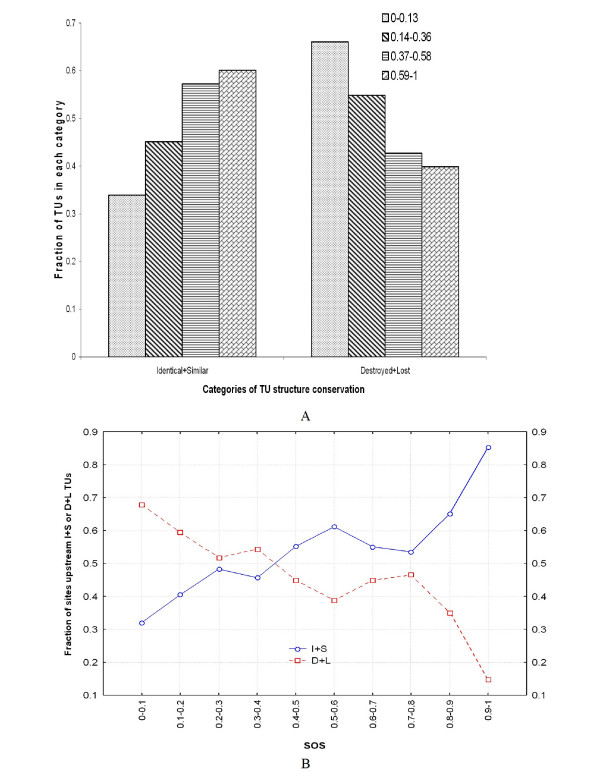
**Relationship between the conservation of a site and the conservation of the TU it regulates**. A) Distribution of TUs in eight categories according to the conservation of their structure (with respect to their *E. coli *orthologs) and the conservation of regulatory sites upstream them. B) Fraction of sites that fall upstream TUs with either Identical or Similar or Destroyed or Lost structures. Sites are grouped in categories of increasing SOS values.

Then, 8 groups of TUs were formed by merging both classification systems. The TUs in each of the four intervals of SOS of their regulatory sites were divided into two classes: those with Identical or Similar structure (I+S), and those with Destroyed or Lost structure (D+L). Figure 5A shows the distribution of the TUs into these 8 groups. The bias in the distribution of TUs between the I+S and D+L groups is also illustrated in Figure 5B, for increasing values of SOS of their regulatory sites.

We calculated an I+S/D+L ratio for each SOS interval, and assessed the statistical significance of the observed ratios with respect to random associations between TUs and regulatory sites. The results (presented in Table 4) show that there is a statistically significant relationship between the conservation of a site and the conservation of the structure of the TU that it regulates, across the organisms of this group. For instance, regulatory sites with SOS between 0 and 0.13 have a probability of only 0.34 of occurring upstream TUs with structures that are either Identical or Similar to their orthologous TU in *E. coli*. On the other hand, sites with SOS between 0.6 and 1 are located upstream Identical or Similar TUs with a probability of 0.6. These results are very similar to those obtained by a previous study that used a simpler equation and less data to calculate the SOS [5]. Furthermore, the assessment of the statistical significance – introduced here – reveals that the biases to more or less conserved TUs observed for each group of sites are highly significant, as shown by the p-values of the ratios, which range from 2.5*10^-69 ^to 1.35*10^-44^.

**Table 4:** Ratios of categories of TUs structure conservation for four intervals of SOS.

SOS intervals	I+S/D+L Ratio	Z score (1000 randomizations)	p-value
0–0.13	0.51	14.01	1.35*10^-44^
0.14–0.36	0.82	14.1	3.8*10^-45^
0.37–0.59	1.34	17.6	2.5*10^-69^
0.6–1	1.51	15.3	7.7*10^-53^

**Figure 6:**
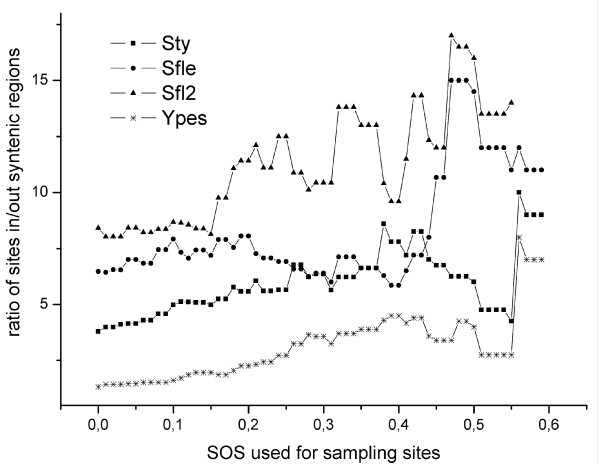
**Ratio of conserved regulatory sites that lie inside or outside conserved syntenic regions**. Ratios of Eco regulatory sites conserved in Sty (squares), Sfle (circles), Sfl2 (triangles), Ypes (stars) are shown.

We also looked at the conservation of regulatory sites in connection to the conservation of gene order between genomes. We counted the number of *E. coli *sites that are conserved in each of the other genomes and separated them into two groups: conserved sites occurring inside conserved syntenic groups of genes and conserved sites occurring outside conserved syntenic groups. To assess whether a bias exists for conserved sites to preferentially appear in one of these two locations and whether this trend correlates in any way with the degree of conservation of regulatory sites across the 30 genomes, we counted sites with SOS equal to or higher than an increasing cutoff value (see above) and calculated a ratio of the two aforementioned groups of sites for each SOS used as cutoff for sampling. Figure 6 shows the values taken by this ratio for sites sampled with increasing SOS cutoff values for pairs of genomes formed by *E. coli *and either Sty, Sfle, Sfl2 and Ypes; Stm has been excluded from the graph for clarity, since its behavior is almost identical to the one observed in Sty; similar trends to the ones shown are observed in all other organisms, but are less clear due to lower number of conserved sites). The graph shows that the number of conserved sites within conserved syntenic groups between *E. coli *and each genome tends to decrease slower than the number of conserved sites outside conserved syntenic regions as higher SOS values are used for sampling the sites. Therefore, regions of conserved synteny between genomes are enriched of sites that are more conserved across genomes. Additional file 5 exemplifies this behavior through the comparison of orthologous genes between Eco and Stm, and Eco and Ypes. Each pair of orthologous genes is represented as a filled circle (when the orthologous of the Eco gene has an incoming interaction that is conserved in the other organism), or as an open circle otherwise. The same trend is observed in other organisms.

## Discussion

To our knowledge, two studies have extrapolated the information on the *E. coli *TRN to other organisms to investigate the evolution of prokaryotic TRNs as a whole [7,19]. Both studies assessed the conservation of *E. coli *(and *B. subtilis*) TFs and regulated genes across more than 100 genomes, showing that TFs are flexibly substituted in the course of evolution. Babu *et al*. [7] found that even network motifs, and the set of global regulators, may change dramatically from one group of organisms to another. (Other works [19,33] have made the same observations regarding network motifs.) Then, they proposed the idea that the free-scale architecture of the TRN evolved in a convergent manner within each group of organisms. On the other hand, several studies that focused on isolated regulons had hinted that within a group of closely related organisms the change of regulatory interactions within the TRN may be at least as important as the substitution of regulatory proteins. In this process (which may be understood as rewiring the TRN), the links between a TF and some of the genes it regulates in the genome of one species are lost in a second (even though both the TF and its regulated genes appear in both) and replaced by regulatory inputs from other TFs. These new TFs need not to be unique of the second species [10-18].

In this study, we aimed at characterizing this process of rewiring the regulatory connections within the TRN that had been noticed for a few isolated regulons. We used the data on putative regulatory sites in 30 gamma-proteobacterial genomes that we had produced using two complementary computational strategies and annotated in Tractor_DB. We intended to investigate not only the genes that belonged to a given *E. coli *regulon and which had apparently left that regulon in another organism but if possible, we wanted to identify the TF(s) that appear to regulate the orthologous genes in that second species. Furthermore, we intended to assess the influence of this change in regulatory connections on structures of higher complexity within the network, i.e., network motifs and the free scale architecture.

Moreover, we wanted to evaluate how this process of rearranging regulatory interactions is reflected on the physical structure of the bacterial chromosome. Since the TU is the basic structure responsible for the spatial proximity of all the sequences necessary to accomplish the correct control of the transcription of a group of genes, we decided to search for changes in the structure of orthologous TUs that might correlate with changes on regulatory input interactions of the genes that form them.

### Robustness of the data stored in Tractor_DB

The scarcity of experimental data on regulatory interactions has been a limitation for comparative analyses that aim at understanding the evolution of TRNs. Manually curated experimental data are available for very few model organisms. Among the prokaryotes *E. coli *K12 [8] and *B. subtilis *[9] have the best known and better annotated networks. To assess the evolution of diverse gamma-proteobacterial regulons, several studies over the past decade have employed computationally predicted regulatory sites. Nevertheless, the use of computationally produced data must always be carefully considered when used as the starting point of biological analyses.

In the case of our study, for example, regulatory sites labeled as **lost link **and **other regulon **may indeed be artefacts of the computational approaches taken to predict orthologous regulatory sites. The fact that such methodologies fail to recognize an ortholog of an *E. coli *regulatory site in another organism (or assign the site to another TF) may be caused by genetic drift (divergence between sequence motifs recognized by orthologous TFs). Also, since the recognition of regulatory sites by TFs is aided by protein-protein interactions, the absence of a sequence strong enough to be identified by a weight matrix does not guarantee that the TF will fail to bind to the regulatory region.

To evaluate the robustness of the data stored in Tractor_DB we compared the results obtained by the methodology of statistical models (Additional file 1) used to predict putative regulatory sites in *E. coli *with experimentally known regulatory sites in this organism, obtained from RegulonDB [8]. Relatively high values of sensitivity and specificity – calculated using the definitions by Benitez-Bellon *et al*. [34] – were achieved by this methodology.

To assess the validity of our computational predictions in other organisms (where sensitivity and specificity cannot be calculated), we compared the *E. coli *experimentally verified TRN (annotated in RegulonDB [8]), and the *E. coli *and *S. typhimurium *reconstructed TRNs with data from gene expression experiments. We compared the expression profiles of co-regulated (or putatively co-regulated) *E. coli *and *S. typhimurium *gene pairs. We found that the strong trend of co-expression observed for experimentally verified co-regulated *E. coli *gene pairs may also be recognized for *E. coli *and *S. typhimurium *computationally predicted co-regulated gene pairs (Additional file 6). As more high quality gene expression data is incorporated into microarray databases, this validation might be extended to other organisms.) These results strongly support the validity of computationally reconstructed TRNs stored in Tractor_DB.

The identification of putative regulatory sites applying a comparative genomics approach based on regulatory information extracted from *E. coli *could in principle reproduce these starting data and create the false impression that gamma-proteobacterial TRNs are very similar. We have used the term circularity to denote this behavior of computationally predicted regulatory binding sites. We have previously tested the data stored in Tractor_DB for this bias [5], and found that orthologous regulons in Tractor_DB differ in gene content between organisms, while they share a common set of genes (the regulon core). Erill *et al*. [13] have characterized this behavior for LexA.

Different TFs present different binding affinity distributions [2,5], which might create another possible bias in our data. These differences might reflect on differences in the information content of statistical models built to identify putative regulatory sites. Weaker models would identify larger number of false positive sites within orthologous regulatory regions, resulting in falsely high SOS values. We tested the hypothesis of a correlation between the SOS of *E. coli *regulatory sites and the scores of their orthologs (calculated by the statistical models used to identify them) that may have arisen as the result of the aforesaid problem. The results in Additional file 7 show that there is no distinguishable trend that relates high SOS values with low sites' scores as would be expected if weak statistical models would have biased SOS values by increasing the number of false positive orthologous regulatory sites.

### Rearrangements of regulatory links are a source of flexibility for the TRN

Babu *et al*. [7] and Lozada-Chavez *et al*. [19]found that the replacement of subsets of *E. coli *and *B subtilis *TFs and (to a lesser extent) structural genes is probably crucial for the adaptation of different evolutionary groups to diverse environmental conditions. This process may be described as the substitution of the TRN "hardware" [20]. In this study, we found that while the replacement of subsets of TFs may be important for the adaptation of organism in different groups to diverse lifestyles, the role played by rearrangements of regulatory connections between conserved TFs and regulated genes in the adaptation of closely related organisms should not be ruled out.

Furthermore, Babu *et al*. [7] showed that TFs and structural genes that are associated in a network motif in *E. coli *do not tend to appear associated in the networks reconstructed in other organisms with a frequency that is higher than that expected by chance. However, our results suggest that if the three genes that form the FF motif in *E. coli *co-occur in another genome the probability that the regulatory links between them are conserved (shown by the values of correlation of their SOS) will be greater than the probability of correlated conservation of a triad of randomly chosen regulatory links. And this trend is also stronger than the one observed for pairs of co-regualted genes, although the latter is also statistically significant when compared to randomly sampled nodes. This observed trend may be a consequence of the dynamical properties that motifs confer to the regulation of individual structural genes [4,25]. Interestingly, some of the highest SOS values correspond to ALs, i.e., TFs that regulate the expression of their structural gene. This means that if a given TF is autorregulated in *E. coli *its ortholog in another gamma-proteobacteria it will probably be autorregulated as well.

The fact that small regulatory circuits as ALs are conserved more often than would be expected from the conservation of individual regulatory interactions – and that regulatory interactions involved in FF motifs are more correlatedly conserved than triads of randomly chosen interactions or pairs of co-regulated genes – suggests that some of these features might be favored by natural selection within a group of closely related organisms once they appear in their common ancestor. In other words, even though the free-scale architecture of TRNs may show converging evolution in different groups of organisms, some of these elements that are present in the TRN of the common ancestor of the group might be preserved by natural selection, while the adaptation of various members of the group to slightly changing lifestyles may be accomplished through changes in regulatory connections, a process that may occur in a short period of time, compared to the substitution of a subset of regulatory proteins.

### Genome rearrangements may contribute to the interchange of regulatory sites

Analysis of the conservation of the structure of TUs showed a trend for conserved sites to be located upstream conserved TUs. On the other hand, regulatory interactions conserved in few genomes involve genes within TUs whose structure is less conserved. This bias suggests that rearrangements of the bacterial chromosome that affect the structure of TUs may be related to the rewiring process observed in the regulatory network. Itoh *et al*. [32] pointed out that when a TU is broken, one of the resulting fragments retains the original regulation (remains close to its regulatory region) while the other fragment(s) by approaching a new regulatory region – through rearrangements – may either retain the same regulation or gain regulatory input from a different TF or set of TFs. Moreover, we found that conserved regulatory sites tend to involve genes that occur within conserved syntenic groups, and that this trend increases when analyzing sites with higher SOS. That is, interactions that are conserved across a higher number of organisms show a stronger trend to affect genes within conserved syntenic groups. Therefore, we raise the hypothesis that rearrangements of genetic material resulting from inversions [19] (and possibly horizontal gene transfer) may have affected the regulation of individual genes. On the other hand, sets of genes whose order in the chromosome is not disrupted (i.e.: conserved syntenic groups) would have maintained their regulatory interactions. Thus, these rearrangement events have contributed to the rewiring of the regulatory network. Nevertheless, both types of events – when affecting large portions of chromosomes that contain both TFs and their regulated genes – would have preserved the spatial proximity between TFs and their regulated genes, as has been observed by Menchaca-Mendez *et al*. [6].

Nevertheless, the importance of orthologous regulatory sequence divergence may not be overlooked when analyzing the causes of network rewiring. We observed that as much as 46% (Paer) to 77% (Ecoo) of all genes that receive interactions from TFs that differ from those that regulate their orthologs in *E. coli *occur in TUs that are identical or similar to their Eco orthologs. Therefore, the difference in regulation for these instances may only be explained by the divergence of the orthologous regulatory regions that may affect the recognition by the model that represents the binding site of the orthologous TF.

## Conclusion

While at large evolutionary scales, the main source of variation of the regulation of individual genes may be changes in the repertoire of TFs (by changing in gene content), at smaller scales, the shift of individual regulatory interactions – or rewiring of the network – between existing TFs and structural genes may be also an important source for this modification. The rewiring observed at the most basic level of the regulatory network occurs mainly by two processes: rearrangements of genetic material and divergence of orthologous regulatory regions. The rearrangements that occur in bacterial chromosomes at this evolutionary scale-mostly inversion or horizontal gene transfer events – alter the structure of some TUs but leave the order of genes in certain pieces of the chromosome unchanged. At this evolutionary scale, the connections that are part of motifs tend to be more correlatedly conserved than triads of randomly chosen regulatory links or pairs of co-regulated genes. Moreover, the rewiring process does not affect the global properties of the graph, such as the connectivity distribution. This preservation of connectivity distribution takes place without modifying the set of global regulators, suggesting that only fine-tuning of the regulatory network occurs within this group of closely related organisms, in the process of divergence from a common ancestor.

## Methods

We determined orthology relationships between gene pairs using the BLAST Bi-Directional Best Hits (BBH) approach [35]. Briefly, two genes were considered orthologs if each gene identified the other as its best hit in the bi-directional blasts satisfying two conditions: the e-values of both hits were equal to or smaller than 10^-15 ^and sequence coverage was at least 60%. These orthology relationships were then extended to other components of the regulatory machinery. We defined **orthologous TUs **as those that share at least one pair of orthologous genes. **Orthologous regulatory regions/sequences **are defined as those extending from -400 to +50 with respect to the first translated nucleotide of orthologous TUs (note that according to this definition, a TU in organism A may have more than one orthologous TUs in organism B). Putative regulatory sites that occur at ortholgous regulatory regions, and are predicted to be recognized by the same TF were labeled as **orthologous regulatory sites**. As mentioned above, we worked with regulatory sites stored in Tractor_DB that were identified using either statistical models or pattern matching [31,36,37]. A thorough description of the methodologies used to produce these computationally predicted regulatory sites is provided in Additional file 1.

In this work, regulatory site conservation is used as synonymous to conservation of a regulatory interaction, although two conserved sites may in practice correspond to a single conserved interaction (when multiple binding sites for the same TF affecting the same gene are conserved). As Table 1 shows, our starting dataset contained a varying number of interactions for the 30 genomes included in the study (from 6 in *X. axonopodis *and *M. capsulatus *to 2118 in *E. coli *K12), with a mean of 446 interactions per genome.

### Variability of regulatory interactions within regulons

We used regulatory interactions identified in *E. coli *as a template to assess the variability of regulatory interactions of each individual regulon across all the organisms included in the database. Putative orthologous interactions – or their absence – in each of the other twenty-nine genomes were classified into four categories. Interactions established between a pair of orthologous TFs and a pair of orthologous structural genes were labeled as **conserved**; a pair of orthologous genes regulated by non-orthologous TFs were included in the **other regulon **category; when an ortholog of an *E. coli *gene with a known input regulatory interaction has no identified regulatory site, we considered this a **lost link**; an *E. coli *gene without an identified ortholog in the other organism was classified as **lost gene**. Figure 1 illustrates this classification scheme.

### Site Orthology Score (SOS)

The orthology score of a site (SOS) assesses the conservation of an *E. coli *regulatory site across the other 29 genomes. Let *a*_*i *_be a binary coefficient that takes value 1 if an orthologous site exists (a putative regulatory site predicted to be recognized by the orthologous TF) in the organism *i *and 0 otherwise (note that *a*_*i *_will take value 1 if at least an ortholog to an *E. coli *site exists in genome i, regardless of the exact number of original sites, orthologous sites and their precise positions relative to each other). Let *f*_*i*-*Eco *_be the fraction of genes of the organism *i *with orthologs in *E. coli*. (*f*_*i*-*Eco *_is a measure of the similarity of a given genome to *E. coli*, and is defined for all 29 pairs formed by *E. coli *and any other organism.) Let then *b*_*i *_be a binary coefficient that takes value 1 if an orthologous site is expected to appear in the organism *i *and 0 otherwise. (Orthologous sites are expected to appear in the genome of *i *if and only if an orthologous TF and at least an orthologous TU of the *E. coli *TU upstream which the site is located exist in the genome of organism *i*.) The SOS may be expressed as:

$$SOS=\frac{\sum_{i=0}^{28}a_{i}(1-f_{i-Eco})}{\sum_{i=0}^{28}b_{i}(1-f_{i-Eco})}.$$

The numerator of the equation counts the number of organisms where orthologous sites are found, weighing the contribution of each organism to the SOS with an estimator of the phylogenetic distance between organism *i *and *E. coli *(one minus the fraction of genes from *i *that have orthologs in *E. coli*). Therefore, the contribution of an organism to the SOS is greater the smaller the fraction of its genes with orthologs in *E. coli*. On the other hand, the denominator computes the highest number of organisms where orthologous sites are expected to occur under the conditions of the strategies used to identify putative regulatory interactions – i.e., an orthologous site may only be found in organisms where an orthologous TF and at least one orthologous TU exist – and normalizes the SOS. The SOS of an *E. coli *site is thus the fraction that results from counting the genomes with an orthologous site (weighed by a measure of their phylogenetic distance to *E. coli*) and dividing them by the sum of all genomes with an expected orthologous site (weighed in the same manner). It is thus a normalized score that reflects the conservation of a site within the set of the 29 genomes included in this study. The SOS does not include the score of putative regulatory sites (assigned by statistical models). Nevertheless, by considering only sites scoring above estimated cutoffs by the predictive approach (see Additional file 1) it indirectly takes into account the similarity of a putative site to the statistical model, and subsequently weighs the significance of a regulatory site in a way that is similar to the Regulogger tool [16].

Since the identification of pairs of orthologous genes may be influenced by a number of factors (horizontally transferred genetic material, paralogy effects that may give rise to the existence of both inparalogs and outparalogs) that could in principle distort the fractions of orthologous genes calculated, we tested the influence of the chosen measure of the evolutionary distance on the distribution of the SOS. We recalculated all the SOS of *E. coli *regulatory links using a different measure of phylogenetic distance. We employed the same measure of distance used by Lozada-Chavez *et al *[19], based on the distances between the nodes of a phylogenetic tree estimated from a set of carefully filtered proteins. We found that the SOS calculated using the fraction of orthologous genes as measure of phylogenetic distances correlates fairly well (R^2 ^= 0.68) with those calculated using the more rigorous measure described in the cited paper. Therefore, a study carried out using a more rigorous measure of phylogenetic distance would most likely arrive to very similar results (see Additional file 8 for details).

The SOS takes values between 0 and 1. A site with SOS 0 has no orthologous sites, and a site with SOS 1 has orthologs in all the organisms where an ortholog is expected given the aforementioned conditions.

We presented a previous version of the SOS in a study that included seven organisms [5]. Instead of being normalized to the estimation of the maximum score of the site, it was normalized to the highest SOS value found among all sites in the entire *E. coli *network. The current improvement produces a more realistic SOS for each site by constraining the maximum expected SOS to the contribution of the organisms where orthologous sites may actually occur.

(The Additional file 9 exemplifies the calculation of the SOS for the FruR binding site upstream the FruB-FruK-FruA TU.)

### Motif conservation

We assessed the conservation of the Auto-regulatory Loop (AL) and the Feed Forward (FF) motifs from *E. coli *across the other 29 organisms with respect to the entire network. In the AL motif, a TF regulates the transcription of its own gene. In the FF motif two TFs, X and Y regulate the expression of gene A: X regulates the transcription of A both directly and indirectly through the regulation of the transcription of the gene coding Y [20,21]. In our starting data set 333 regulatory interactions formed 111 FF motifs and 28 AL motifs wtih TFs regulating transcription of their own genes. The number of motifs in our data set is smaller than the total number of motifs in the *E. coli *regulatory network [7], because Tractor_DB contains putative regulatory sites for only 84 *E. coli *TFs, due to the intrinsic limitations of the computational strategies employed to identify putative regulatory sites, i.e: some TFs do not have enough known binding sites to build a statistically significant weight matrix, and in average only 47% of *E. coli *genes have orthologs in the other 29 genomes.

### Conservation of AL motifs

We calculated the mean SOS of the interactions involved in the 28 AL motifs in the *E. coli *network (M_AL_). Then, we sampled 1000 random groups of 28 interactions from the *E. coli *network and calculated the mean SOS for each group. The mean and standard deviation of the 1000 random means were subsequently calculated. These measures were then used to assess the statistical significance of the M_AL_, through the calculation of the corresponding Z score and p-value.

### Conservation of FF motifs

We calculated correlations for the three pairs of regulatory interactions (their SOS) that form FF motifs. (Actually, we took the 111 FF motifs in the *E. coli *network and calculated the Pearson Correlation Coefficient of the three pairs of 111 sets of SOS of homologous interactions.) First, we calculated the mean SOS values corresponding to the TF_X_-TF_Y_, the TF_X_-A, and the TF_Y_-A interactions from all the FF motifs in the network. Then, we calculated the standard deviation value of these three interactions and the covariance of the three possible pairs of interactions (TF_X_-TF_Y_/TF_X_-A, TF_X_-TF_Y_/TF_Y_-A and TF_X_-A/TF_Y_-A) in the set of 111 FF motifs. Finally, we calculated the correlations of the mean SOS values of the three interaction pairs. For instance, we calculated the correlation between the SOS of all the TF_X_-TF_Y _interactions and all the TF_X_-A interactions (cor_1_) using the equation

$$cor_{1}=\frac{\sum_{i=0}^{N}\left(SOS_{XY_{i}}-M_{XY}\right)\left(SOS_{XA_{i}}-M_{XA}\right)}{\left(\sqrt{\sum_{i=0}^{N}{\left(SOS_{XY_{i}}-M_{XY}\right)}^{2}}\right)\left(\sqrt{\sum_{i=0}^{N}{\left(SOS_{XA_{i}}-M_{XA}\right)}^{2}}\right)},$$

where *N *is the number of TF_X_-TF_Y _(or TF_X_-A) regulatory interactions, i.e., 111; *SOS*_*XYi *_and *SOS*_*XAi *_are the SOS of the *i*-th TF_X_-TF_Y _interaction and the *i*-th TF_X_-A interaction, respectively; and *M*_*XY *_and *M*_*XA *_are the mean SOS of the TF_X_-TF_Y _interactions and the TF_X_-A interactions, respectively. (Correlations between the other two pairs of interactions were calculated analogously.)

Since the denominator of the SOS computes the number of organisms for which a site orthologous to the *E. coli *original site appears (i.e., organisms with orthologous TFs and structural genes to those involving the *E. coli *regulatory interaction) this correlation does not assess whether a trio of *E. coli *genes involved in a FF motif co-occur in other organisms. Instead, it assesses whether a trio of *E. coli *regulatory links appear together in other organisms that bear orthologs for the three genes.

To assess the statistical significance of the correlations calculated for each pair of interactions, we randomly sampled 1000 groups of 111 triads of interactions from the *E. coli *network, and calculated the corresponding 1000 triplets of correlations. Then, we calculated the mean and standard deviation of each of these three random sets of correlations and the Z score and p-value corresponding to the three true correlation values.

We also extracted the 4089 pairs of computationally predicted *E. coli *co-regulated genes from Tractor_DB (i.e.: all pairs of genes predicted to be regulated by the same individual TF). We then calculated the Pearson correlation coefficient of the SOS corresponding to the regulatory links that connect the TF with the genes that form each pair. The calculation of the Pearson correlation coefficient and the assessment of its statistical significance were carried out as described above for the FF motif (in this case only pairs of interactions were randomly sampled to calculate the Z score).

### Conservation of regulatory sites and TU structure

The conservation of the structure of each *E. coli *TU was assessed using the qualitative classification proposed by Itoh *et al*. [29]. Briefly, an orthologous TU is classified as Identical if its structure, number of genes and order are the same than the *E. coli *TU at the center of the analysis; as Similar if the structure is only partly conserved (allowing translocations, deletion and up to two insertions); as Destroyed if at least two orthologous genes of the *E. coli *TU are found, but they belong to different orthologous TUs; and finally, as Lost when at most one ortholog to the genes of the *E. coli *TU is found.

We labeled each orthologous TU using this classification system, and associated the label of each TU to the SOS of the regulatory site(s) found at their regulatory regions. TUs were clustered into four groups according to the SOS of their regulatory sites: TUs with SOS between 0 and 0.13, TUs with SOS between 0.14 and 0.36, TUs with SOS between 0.37 and 0.59 and TUs with SOS between 0.6 and 1 as in Espinosa *et al*. (2005) [5].

To find out whether the resulting association is statistically significant with respect to all possible associations of SOS and TU structure conservation we randomized the association between TUs and regulatory sites, keeping both SOS frequencies and the frequencies of the four categories of TUs structure conservation. This randomization was iterated 1000 times and for each randomization we calculated the resulting ratios of Identical and Similar TUs to Destroyed and Lost TUs in each group of SOS. Finally, the mean and standard deviation of each SOS group ratio was calculated, as well as the corresponding Z scores and p-values.

## List of Abbreviations

SOS: Site orthology score; TU: Transcription unit; TF: Transcription factor; TRN: Transcriptional regulatory network.

## Authors' contributions

AGP carried out SOS calculations and motifs conservation assessment, participated in the conception and design of the study, in TUs conservation assessment, contributed to the discussion, and drafted the manuscript. EGG participated in the design of the study, and in TUs conservation assessment. VEA participated in the conception of the study, and in TUs conservation assessment, and helped drafting the manuscript. ATRV and JC-V coordinated the study, contributed to results, discussion, and helped drafting and revising the manuscript. All authors read and approved the final manuscript.

## Supplementary Material

Additional file 1 M1: Approach to prediction of putative TF binding sites Click here for file

Additional file 2 R6: The process of disappearance/appearance of regulatory links Click here for file

Additional file 3 R1: Specificity and sensitivity of the identification of putative regulatory sites stored in Tractor_DB Click here for file

Additional file 4 T1: Distribution of the SOS (maximum value, mean value, and standard deviation) for each regulon and globally within the network Click here for file

Additional file 5 R4: Conservation of regulatory sites and chromosome vicinity of their target genes Click here for file

Additional file 6 R5: Assessing the quality of reconstructed TRNs using expression data Click here for file

Additional file 7 R3: SOS values of E. coli regulatory sites against mean scores of their orthologous sites Click here for file

Additional file 8 R7: Recalculation of the SOS using a different measure of phylogenetic distance Click here for file

Additional file 9 R2: Calculation of the SOS of a regulatory link Click here for file
