# Supplementary material for: Impact of Transcription Units rearrangement on the evolution of the regulatory network of gamma-proteobacteria
Source: BMC Genomics. 2008 Mar 17;9:128. doi: 10.1186/1471-2164-9-128 (PMC2329645; doi:10.1186/1471-2164-9-128)
Supplement: Additional file 1 — M1: Approach to prediction of putative TF binding sites [file 1471-2164-9-128-S1.pdf]

M1: Approach to prediction of putative TF binding sites

A) Predicting sites using statistical models

Summary

We inherited three main ideas from Tan's methodology (3): 1) filtering the training set in two successive steps, as explained below, to eliminate possible weak binding sequences; 2) cutoff selection based on the occurrence of putative sites in and outside TUs; and 3) filtering the final set of predictions using orthology information and knowledge of clustering of genes into TUs. The latter idea requires a) a reliable prediction of the genome organization in TUs for all the organisms under study -we used predictions described in (1)-; and b) a reliable methodology for orthology searching between genomes. Orthology relationships were searched using the definition by Huynen and Bork (2): two genes from different organisms are orthologs if they are the best bi-directional blast hits (BBHs). For a thorough description of these issues, see (3). We used two algorithms to build the models: the CONSENSUS (4), and the Gibbs-SAMPLER (5). The different steps of this methodology were implemented using *ad hoc* PERL scripts, and the MySQL relational database.

Also, this methodology implicitly assumes that if TF A of *E. coli* has an ortholog in another organism, the two will recognize roughly the same DNA motif. Although this is not necessarily true, the failure of this assumption does not have any major consequences: if the DNA binding motif of a TF significantly differs from the DNA binding motif of its *E. coli* ortholog, the original statistical models (see below) that are built (or enriched) from *E. coli* DNA binding sequences will simply fail in recognizing its putative binding sites.

1. Building original models with CONSENSUS and preparing the training set

Original training sets include sequences extracted from RegulonDB, v.4.0 (6). A training set was built for each TF with at least one known binding sequence in *E. coli* **(102)**. For TFs with a training set larger than 25 sequences, only *E. coli* known binding sequences were used to build the original model. In order to populate the training sets of those factors with less than 25 known binding sequences, all the sequences upstream TUs (strictly non-coding) of all other organisms having at least one ortholog to an *E. coli* gene known to be regulated by the given TF were included in the input for CONSENSUS. We built weight matrices only for TF's with training sets larger than 4 sequences **(83)**.

To select the appropriate site length to build each TF's model, the following was done: different matrix lengths were assayed, ranging from 16 to 30 nucleotides and, for every length, two matrices were built, one using a symmetric model (by forcing the algorithm to include within the model the reverse complement of the sequences in the training set) and a second one where only original sequences were included. The appropriate length was selected as the one that produced the matrix with the lowest expected-frequency (4).

The training sets were filtered twice as proposed by Tan *et al.* (3) to eliminate possible weak binding sequences. In the first filtering step only the site that produce the best score in each TU when aligned to the site model remained in the training set. The

model was then rebuilt with only those sequences which were aligned against this second model. The second step eliminated all sequences that scored below the mean minus one standard deviation in this alignment.

As expressed above, we also used Tan's ideas regarding cutoffs selection, but with slight variations. Briefly, according to these ideas cutoff values are defined after searching for putative binding sites within a given genome using weight matrices built using filtered training sets. Increasing cutoff values are assayed, and putative binding sites obtained for each cutoff value inside TUs and within non-coding regions are recorded separately and counted. For lower cutoff values, putative binding sites are expected to occur much more frequently within TUs (since prokaryotic genomes are enriched in coding regions). On the other hand, known binding sites occur almost exclusively outside TUs; hence, as cutoff values increased, the number of putative sites recorded inside TUs should tend to decrease faster than the number of putative sites recorded within non-coding regions, therefore increasing the fraction of putative sites within non-coding regions. One can, then define cutoff values with a reasonable expectation of the rate of false positives to be obtained in the search for putative binding sites.

Two cutoff values were defined: a strong one corresponding to the score value where all predictions occurred outside TUs, and a weak value, that corresponded to the score for which at least 50% of all predictions fell outside TUs. We accepted the second value to be the weak cutoff, only if it was greater than or equal to the weakest sequence within the filtered training set, that is, at least the mean minus one standard deviation of all the sequences in the training set after all duplicate sequences for a given TU had been filtered out. If this was not the case, the weak cutoff value was set to the score of the weakest sequence within the filtered training set. Those TFs for which the training set was reduced to less than four sequences at any filtering step were eliminated from the study.

2. Building original models with SAMPLER and preparing the training set

Original training sets were built following the same rules described for CONSENSUS, but more parameters were assayed to tune model building. There are three parameters influencing the stringency of the multiple local alignments (7): the number of models that the SAMPLER is set to search for, the minimum length of sites and the Expected Number of sites that each sequence contributes to the model.

In our case we restricted the searches to a single model, since we included within the training set all the known sequences of each respective TF. We iterated the Gibbs-Sampler varying the expected number of sites, n for all Factors and set n equal to two thirds of the number of sequences in the training set as a general rule, because we found it was an acceptable value for all TFs under study. We have also run the program for all factors with minimum site length varying from 16 to 24 bp.

The Maximum *a posteriori* Probability (MAP) (5) was used as an indicator: in the case of TFs which produced alignments with the best MAPs corresponding to even lengths, the minimum site length was set to 20 base pairs; when odd alignments produced higher MAP values, 19 base pairs was chosen as the minimum site length. In those cases where a clear decision could not be made, the minimum site length was set to 20.

A major difference between the two algorithms is that for Gibbs-SAMPLER cutoffs were not calculated *a priori*, before the genome was scanned using the model.

Instead, the counting of predictions falling in and outside TUs is done while the genome is being scanned, with decreasing values of *-logE* (logarithm of the expected value; see below) and the scanning ends when the same number of predictions is counted in and outside TUs.

3. Obtaining the sets of putative new binding sites with CONSENSUS

After the model for each TF had been built as described before and the two cutoff values had been selected, the regions upstream all TUs, ranging from -400 to +50 nucleotides, the region where almost all known regulatory sites in σ^{70} promoters occur (8), of the seventeen genomes included in version 1.0 of the database were searched for putative new binding sites (*Escherichia coli* K12, *Haemophilus influenzae, Salmonella typhi, Salmonella typhimurium* LT2*, Shewanella oneidensis, Shigella flexneri* 2a*, Vibrio cholerae, Yersinia pestis* KIM, *Buchnera aphidicola*, *Pseudomonas aeruginosa*, *Pseudomonas syringae*, *Pasteurella multocida*, *Pseudomonas putida* KT2440, *Vibrio parahaemolyticus*, *Vibrio vulnificus* CMCP6, *Xanthomonas axonopodis*, *Xylella fastidiosa*). The matrix built using CONSENSUS was aligned against these regions using PATSER (4), setting the lower threshold of the program to the weak cutoff value.

4. Obtaining the sets of putative new binding sites with SAMPLER

We used the DSCAN software (7) to scan the genome in a database built as follows: a set of upstream sequences ranging from at most -400 to +50 bp, keeping the negative portion of the sequences strictly extragenic, -i.e., there are no overlaps with neighbor coding regions; and a second set containing the sequences of TUs, from position +50 of the first gene to the last base of the last gene. We then concatenated these two sets which have no overlapping sequences.

We did this because of the statistical nature of the calculations performed by DSCAN. For each set of sequences DSCAN returns the expectation value (the E-value is the expected number of sites of equal or higher score that would be retrieved with the same search performed on a randomly constructed database of the same size) and the probabilities (p-values) of finding a better scoring segment (better than the higher scoring segment in the sequence) in a random sequence of the same length (7).

5. Orthology filtering

The sets of putative sites produced by CONSENSUS and Gibbs-SAMPLER were merged and subsequently filtered using orthology information. Two sites predicted by CONSENSUS and SAMPLER were considered different if they did not overlap more than 50%, otherwise they were considered a redundant prediction, and as such, filtered out. A prediction was marked as "reliable" if it occurred within the region upstream an *E. coli* TU that had at least a gene with one or more orthologs in the other genomes and the region upstream the TUs where those orthologs were located had a prediction for the same TF. The hypothesis beneath this idea is that regulation systems tend to be conserved during evolution. Alternatively, a putative site in any genome with a score above the strong cutoff with no orthology information supporting it was also marked as "reliable", given its high similarity to the model and taking into account the fact that a fraction of an organism's ORFs (at least 30% for *E. coli* with respect to any of the other 7 organisms)

has no recognizable orthologs, so these sites may be involved in the regulation of these unique genes.

6. Rebuilding models

New training sets were built rescuing putative sites predicted for each TF in all organisms. For those TFs that produced more than four putative sites in *E. coli* (after the orthology filtering) and in at least another organism, a training set was built for each separate organism with more than four putative sites. These sets were then used to build a specific model for each organism using CONSENSUS. On the other hand, matrices were not rebuilt for those TFs for which less than four sites were found. Instead, the sequences found for these TFs in the previous step of the methodology were directly included in the set of putative sites produced after rebuilding matrices and were again filtered by orthology information as described below.

The orthology filtering process is very similar to the one already described. The only difference is that filtering is done with respect to each organism. This assures that genes that are not present in *E. coli* but are common to a pair of other organisms can be rescued as members of the same regulon in those two organisms. More complete lists of regulons' members can thus be produced and their properties compared across the genomes under study.

B) Predicting sites using regular expressions

1. Building regular expressions

We transformed each *E. coli* known binding site into a regular expression following these rules: if the site length was even and shorter than 14 nucleotides, the site sequence was expanded at both ends to reach 14 nucleotides (with the center between the two central base pairs); if the site was odd and shorter than 13 nucleotides, the site sequence was expanded at both ends to reach 13 nucleotides (with the center at the central base pair); on the other hand, the site length reported at RegulonDB was respected if the site was longer than 13 (odd) or 14 (even) nucleotides. The reason to take 13 or 14 nucleotides as the minimum length for odd and even sites respectively is that the length of most prokaryotic TF binding sites rages from 14 to 26 nucleotides. After this step, site lengths ranged from 13 nucleotides (DnaA, IHF, IciA, XapR, and CspA) to 61 nucleotides (ArcA).

To perform the pattern matching, we extracted the r-orthologous regions (from -400 to +50 with respect to the first base of the TU, the region where most TF binding sites are known to occur). We searched the occurrences of the *E. coli* regular expressions of each TF in all orthologous regulatory regions and allowed in the process of pattern matching the lowest number of mismatches that recovered a number of matches of the same order that the number of *E. coli* known sites of the TF, a cutoff selected to reduce the number of spurious matches. No gaps were allowed in the pattern matching, taking into account the high sequence identity between orthologous regulators in these organisms (3, 9). We allowed no more than 8 mismatches for any TF.

2. Assessing the statistical significance of predicted sites

Using the CONSENSUS program (4) we built weight matrices for each TF aligning the sequences of original *E. coli* sites and their putative orthologous sites identified by pattern matching. These sequences were extended five nucleotides at each side before introducing them into the program. The idea is that pattern matching may locate a true orthologous site displaced several bases at the left or right: the CONSENSUS algorithm is able to correctly locate the sites by maximizing the log-likelihood of the alignment. The program was run including the reverse-complement sequences into the matrices in the case of dimeric binding TFs.

To asses the statistical significance of putative orthologous sites we used the idea developed by Brown and Callan (9) for CRP. Briefly, each predicted orthologous sequence is aligned against the weight matrix that represents the TF binding site and the score of this alignment is termed "real". On the other hand an "expected" score of the predicted sequence is calculated as a result of its alignment against the matrix provided that it mutated following a rate similar to the rest of the non-coding region where it is located. (Mutation rates are calculated from the alignment of the r-orthologous regions.) The idea here is that in an interspecies comparison, true orthologous regulatory sites should be more conserved than the rest of the non-coding regions. (For a thorough description of the statistics carried out in these calculations see 9.) To calculate the real score of putative orthologous sites we aligned them to the corresponding weight matrix using the PATSER program (4), and to estimate the score of the site had it mutated with the same rate that the surrounding non-coding region (expected score) we used the package of programs provided by Brown and Callan in their already cited work, located at www.princenton.edu/ccallan/binding.

C) Expanding the database to 30 organisms

This work reproduces the methodology described for predicting putative binding sites using weight matrices. Briefly, this expansion started by building positional weight matrices from training sets constituted by each TF's known binding sites in *E. coli* and orthologous regulatory regions in other seven organisms (those phylogenetically closer to *E. coli*, excluding *E. coli* O157H7 and *Shigella flexneri* 2a 2457T strains to avoid biasing the matrix), out of the 30 included in the database (see Table 1 for the complete list). Then, after filtering such training sets to eliminate possible weak binding sequences and calculating two cutoff values for each TF, the regulatory regions of all genomes were scanned for putative binding sites using each TF's matrix. The sites thus obtained were filtered using orthology information (all *E. coli* sites without at least one ortholog in at least one of the other 29 genomes are discarded). Finally, a separate matrix was built for each organism from the putative binding sequences retrieved by the first matrix –for all cases with more that four sequences rescued from the filtering process—and the scanning and filtering steps were repeated, with the difference that all possible inter-genome orthology relationships were included in the analysis, and not only those centred at *E. coli*.

The expansion to 30 organisms using the statistical models approach was complemented with the pattern matching (regular expressions) approach as explained above for the

original 17 genomes. We should point out that the filtering process of non-statistically significant orthologous sites (see above) discards an important number of putative orthologous sites in *E. coli* O157 strain, due to the similarity of non-coding regions between its genome and the genome of the K12 strain.

References

1. Moreno-Hagelsieb, G., Collado-Vides, J. (2002) A powerful non-homology method for the prediction of operons in prokaryotes. *Bioinformatics*. **18**: S329-S336.
2. Huynen, MA., Bork, P. (1998) Measuring genome evolution. *Proc.Natl.Acad.SciUSA* **95**:5849-5856.
3. Tan, K., Moreno-Hagelsieb, G., Collado-Vides, J., Stormo, G. A Comparative Genomics Approach to Prediction of New Members of Regulons. (2001) *Genome Research.* **11**:566-584.
4. Hertz, GZ., Stormo, GD. (1999) Identifying DNA and protein patterns with statistically significant alignments of multiple sequences. *Bioinformatics*. **15**: 563-577.
5. Lawrence, CE., Altschul, SF., Boguski, MS., Liu, JS., Neuwald. AF., Wootton, JC. (1993) Detecting subtle sequence signals: a Gibbs sampling strategy for multiple alignment. *Science*. **262**: 208-214.
6. Salgado, H., Gama-Castro, S., Martínez-Antonio, A., Díaz-Peredo, E., Sánchez-Solano, F., Peralta-Gil, M., Garcia-Alonso, D., Jiménez-Jacinto, V., Santos-Zavaleta, A., Bonavides-Martínez, C., and Collado-Vides, J. (2004) RegulonDB (version 4.0): Transcriptional Regulation, Operon Organization and Growth Conditions in *Escherichia coli* K-12". *Nucleic Acids Res.*. **32**: 303-306.
7. Neuwald, AF., Liu, JS., Lawrence, CE. (1995) Gibbs motif sampling: detection of bacterial outer membrane protein repeats. *Protein Sci*. **4**:1618-1632.
8. Gralla J.D., and Collado-Vides J. (1996) Organization and Function of Transcription Regulatory Elements chap. 79 In: Neidhardt F.C., Curtiss III R., Ingraham J., Lin E.C.C., Low K.B., Magasanik B., Reznikoff W., Schaechter M., Umbarger H.E. and Riley M. (eds) Cellular and Molecular Biology: Escherichia coli and Salmonella. 2nd ed. Washington, D.C. American Society for Microbiology pp.1232-1245.
9. Brown, C. T. and Callan, C. G. (2004). Evolutionary comparisons suggest many novel cAMP response protein binding sites in *Escherichia coli*. Proc.Natl.Acad.Sci. USA. **101**, 2404-2409.
