# Supplementary material for: Impact of Transcription Units rearrangement on the evolution of the regulatory network of gamma-proteobacteria
Source: BMC Genomics. 2008 Mar 17;9:128. doi: 10.1186/1471-2164-9-128 (PMC2329645; doi:10.1186/1471-2164-9-128)

R6: The process of disappearance/appearance of regulatory links

Although the classification scheme outlined in the paper to qualitatively assess the process of disappearance/appearance of regulatory links is based on *E. coli*, it illustrates the process of evolutionary divergence of the different gamma-proteobacteria from their last common ancestor. Say a gene X is regulated by TF A in *E. coli* and by TF B in *S. typhimurium*. (In our scheme it would be classified as **other regulon** if both regulatory sites are identified.) This implies that whichever the regulator was in the last common ancestor (see Erill *et al*., 2003 [13] for a discussion on regulon core and the divergence of gene regulation) of these two gamma-proteobacteria, a change has occurred in the regulation of gene X since the divergence of both lines.

This process is more complex when the gene analyzed receives regulatory inputs form different TFs. A gene may be under the regulation of two TFs in one organism and be regulated by only one of them in other genome. We deal with these cases by separating all regulatory inputs received by a gene in *E. coli* and analyzing their states in all other organisms. Likewise, all regulatory inputs not identified in *E. coli*, but recognized in other genomes are separated. The following Figure exemplifies these ideas:

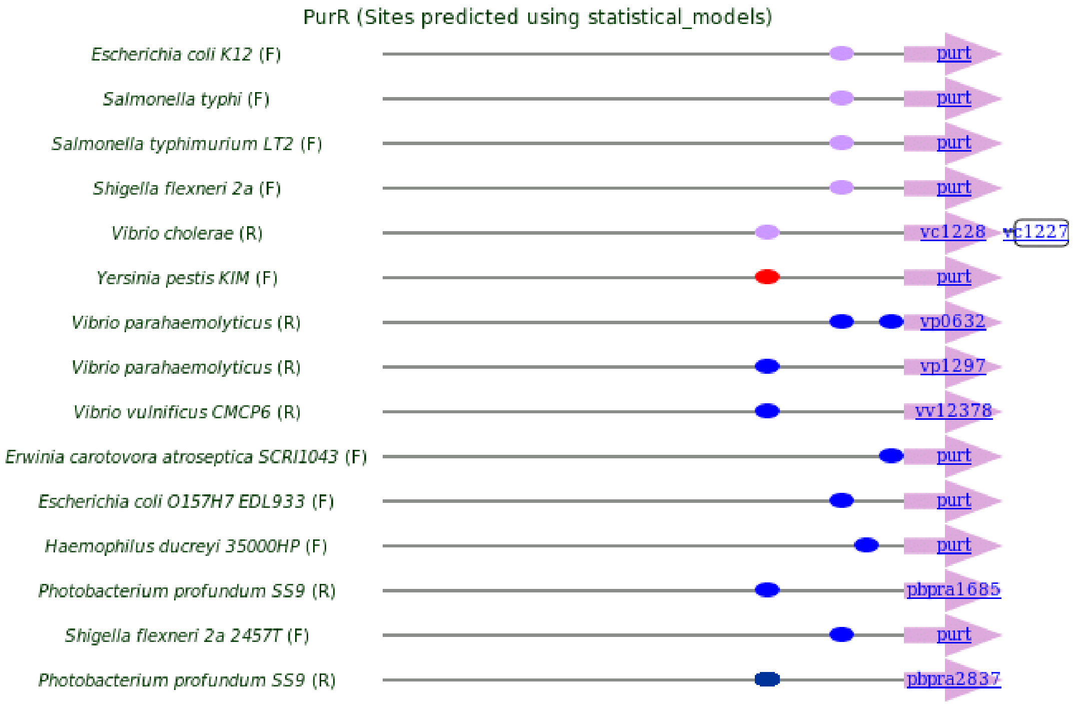

The gene *purt* is predicted to be regulated by PurR in 12 genomes (beside *E. coli*). Therefore, this link is classified as **conserved** by our system in these organisms. On the other hand, it is predicted to be regulated also by LexA in *E. coli*, *S. typhi* and *S. typhimurium*. Thus, this second link is classified as **conserved** in the latter two organisms, and as **lost** in the remaining 10 where the PurR input is conserved. In this case it is reasonable to think that *purt* was originally (i.e., in the common ancestor) regulated by PurR (given the number of descendents where this regulatory link is conserved and the

function of the *purt* product), and that the LexA regulatory input appeared in *E. coli*, *S. typhimurium* and *S. typhi* satisfies particular adaptive needs of these organisms. Nevertheless, whatever the actual change taken place in the regulation of *purt* in the course of the evolution of these groups has been, our system detects its occurrence by multiple pairwise comparisons of the regulatory links found in *E. coli* for a particular gene and those found in all other organisms for its orthologs.

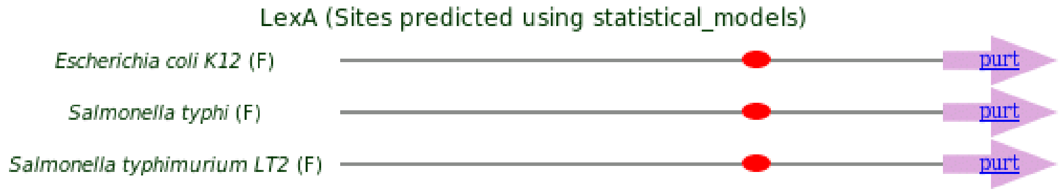

Supplement: Additional file 2 — R6: The process of disappearance/appearance of regulatory links [file 1471-2164-9-128-S2.pdf]
