# Supplementary material for: Impact of Transcription Units rearrangement on the evolution of the regulatory network of gamma-proteobacteria
Source: BMC Genomics. 2008 Mar 17;9:128. doi: 10.1186/1471-2164-9-128 (PMC2329645; doi:10.1186/1471-2164-9-128)
Supplement: Additional file 3 — R1: Specificity and sensitivity of the identification of putative regulatory sites stored in Tractor_DB [file 1471-2164-9-128-S3.pdf]

R1: Specificity and sensitivity of the identification of putative regulatory sites stored in Tractor_DB

Here we present a summary of the results obtained by the identification of putative regulatory sites by the statistical models approach (See Suplementary Material M1). The number of TUs with putative sites identified for each TF in *E. coli* was counted. These results were then compared to the sets of experimentally determined regulatory sites in this organism (stored in RegulonDB, 1), to assess the sensitivity and specificity of the search. These parameters were calculated following the definition by Benitez-Bellon *et al* (2).

Briefly, the sensitivity of the search for putative binding sites for a TF was calculated as the fraction of the TUs with known binding sites that are identified by the search, i.e., the fraction of true positives (corresponding to the ration of columns *Rec.* and *T. set* in Table 2SM). On the other hand, the specificity of the search for a TF was calculated employing the set of TUs with known binding sites for all TFs except the one evaluated in each case. For each TF this set was split into two subsets: TUs for which the search for the given TF fails identifying putative binding sites (true negatives) and TUs for which the search for the given TF identifies putative sites (false positives). The specificity is then calculated as the ratio between the number of true negatives and the sum of true negatives and false positives.

References

1. Salgado H, Gama-Castro S, Peralta-Gil M, Díaz-Peredo E, Sanchez-Solano F, Santos-Zavaleta A, Martínez-Flores I, Jimenez-Jacinto V, Bonavides-Martínez C, Segura-Salazar J, Martínez-Antonio J, Collado-Vides J. (2006) RegulonDB (version 5.0): Escherichia coli K-12 transcriptional regulatory network, operon organization, and growth conditions. *Nucleic Acids Res.* **34**, D394-D397.
2. Bénitez-Bellón, E., Moreno-Hagelsieb, G., Collado-Vides, J. (2002) Evaluation of thresholds for the detection of binding sites for regulatory proteins in *Escherichia coli* K12 DNA. *Genome Biology*, **3(3)**, 0013.1-0013.6.

Table: Number of *E. coli* transcription units where putative binding sites for each TF were found employing the methodology of statistical models. **T. set**: Number of *E. coli* transcription units in the training set**. Rec**: Number of TUs with experimentally known binding sites that are identified by the search; **New**: Number of new putative sites; **Not rec**: Number of experimentally known sites that fail to be identified by the methodology; **Total**: Total number of sites identified by the methodology (experimentally known or not); **Sens**: Sensitivity of the search; **Spec**: Specificity of the search.

TF	T. set	Rec	New	Not rec	Total	Sens(%)	Spec (%)
**Ada**	4	1	6	3	7	25	99.6
**AppY**	2	2	0	0	2	100	100
**AraC**	5	4	4	1	8	80	100
**ArcA**	12	1	6	11	7	8.3	99.3
**ArgR**	7	7	51	0	58	100	96.5
**AsnC**	2	2	0	0	2	100	100
**BetI**	2	2	0	0	2	100	100
**BirA**	2	2	18	0	20	100	99.7
**CadC**	1	1	7	0	8	100	98.6
**CaiF**	2	2	0	0	2	100	100
**CRP**	90	67	412	23	479	74.4	79.6
**CsgD**	2	1	0	1	1	50	100
**CspA**	2	2	3	0	5	100	99.7
**CytR**	4	2	1	2	3	50	100
**DcuR**	2	1	0	1	1	50	100
**DeoR**	1	1	0	0	1	100	100
**DnaA**	3	0	2	3	2	0	99.3
**DsdC**	3	2	2	1	4	66.7	100
**EbgR**	1	1	0	0	1	100	100
**EnvY**	2	1	1	1	2	50	100
**ExuR**	1	1	1	0	2	100	100
**FabR**	1	1	5	0	6	100	100
**FadR**	3	2	3	1	5	66.7	99.6

TF	T. set	Rec	New	Not rec	Total	Sens(%)	Spec (%)
**FhlA**	5	0	1	5	1	0	100
**FNR**	33	21	213	12	234	63.6	84.4
**FruR**	11	8	11	3	19	72.7	99.3
**FucR**	2	2	0	0	2	100	100
**Fur**	9	7	64	2	71	77.8	97.1
**GadW**	2	2	0	0	2	100	100
**GadX**	2	2	0	0	2	100	100
**GalR**	2	1	0	1	1	50	100
**GalS**	2	2	1	0	3	100	100
**GatR**	1	1	0	0	1	100	100
**GcvA**	2	2	14	0	16	100	97.9
**GlcC**	2	2	1	0	3	100	99.7
**GlpR**	4	4	16	0	20	100	98.6
**GntR**	2	2	0	0	2	100	100
**HyfR**	1	1	0	0	1	100	100
**IclR**	1	1	0	0	1	100	100
**IlvY**	2	2	0	0	2	100	100
**LexA**	10	7	20	3	27	70	99.3
**LrhA**	2	1	0	1	1	50	100
**Lrp**	12	7	7	5	14	58.3	99.6
**LysR**	2	2	12	0	14	100	97.9
**MarA**	8	1	0	7	1	12.5	100
**MarR**	1	1	1	0	2	100	100
**MelR**	2	2	0	0	2	100	100
**MetJ**	3	3	19	0	22	100	99.3
**Mlc**	4	2	0	2	2	50	100
**ModE**	6	4	7	2	11	66.7	100
**Nac**	2	1	50	1	51	50	96.2
**NadR**	2	2	1	0	3	100	100
**NarL**	13	7	47	6	54	53.8	97.8
**NarP**	2	1	1	1	2	50	100
**NhaR**	1	1	0	0	1	100	100
**NtrC**	4	4	7	0	11	100	99.6
**OmpR**	6	2	0	4	2	33.3	100
**PdhR**	1	1	0	0	1	100	100
**PhoB**	6	3	13	3	16	50	99.6
**PhoP**	2	2	7	0	9	100	99.3
**PurR**	16	13	7	3	20	81.3	100

TF	T. set	Rec	New	Not rec	Total	Sens(%)	Spec (%)
**RhaR**	1	1	1	0	2	100	99.7
**RtcR**	1	1	1	0	2	100	100
**SlyA**	1	1	1	0	2	100	100
**TorR**	2	2	2	0	4	100	100
**TreR**	1	1	0	0	1	100	100
**TrpR**	3	1	0	2	1	33.3	100
**TyrR**	6	4	7	2	11	66.7	100
**UhpA**	1	1	0	0	1	100	100
**XapR**	1	1	1	0	2	100	100
**XylR**	2	2	0	0	2	100	100
**YiaJ**	1	1	1	0	2	100	99.7
