# Supplementary material for: Impact of Transcription Units rearrangement on the evolution of the regulatory network of gamma-proteobacteria
Source: BMC Genomics. 2008 Mar 17;9:128. doi: 10.1186/1471-2164-9-128 (PMC2329645; doi:10.1186/1471-2164-9-128)
Supplement: Additional file 4 — T1: Distribution of the SOS (maximum value, mean value, and standard deviation) for each regulon and globally within the network [file 1471-2164-9-128-S4.pdf]

**T1: Distribution of the SOS (maximum value, mean value, and standard deviation) for each regulon and globally within the network**

| Regulon | Maximum SOS | Mean SOS | SOS SD |
|---|---|---|---|
| **Ada** | 0.2 | 0.1 | 0.05 |
| **AppY** | 0.23 | 0.14 | 0.05 |
| **AraC** | 0.32 | 0.17 | 0.1 |
| **ArcA** | 0.25 | 0.12 | 0.08 |
| **ArgR** | 0.69 | 0.17 | 0.2 |
| **AsnC** | 0.23 | 0.23 | 0 |
| **BetI** | 0.18 | 0.12 | 0.06 |
| **BirA** | 0.18 | 0.06 | 0.05 |
| **CadC** | 1 | 0.33 | 0.26 |
| **CaiF** | 0.76 | 0.34 | 0.3 |
| **CpxR** | 0.04 | 0.03 | 0.02 |
| **CRP** | 0.54 | 0.11 | 0.09 |
| **CsgD** | 0.44 | 0.44 | 0 |
| **CspA** | 0.2 | 0.08 | 0.07 |
| **CysB** | 0.04 | 0.01 | 0.02 |
| **CytR** | 0.46 | 0.29 | 0.11 |
| **DcuR** | 0.07 | 0.07 | 0 |
| **DeoR** | 0.09 | 0.09 | 0 |
| **DnaA** | 0.29 | 0.28 | 0.02 |
| **DsdC** | 0.18 | 0.09 | 0.09 |
| **EbgR** | 0.08 | 0.08 | 0 |
| **EnvY** | 0.31 | 0.31 | 0 |
| **ExuR** | 0.06 | 0.06 | 0 |
| **FabR** | 0.4 | 0.16 | 0.16 |
| **FadR** | 0.39 | 0.15 | 0.1 |
| **FarR** | 0.38 | 0.22 | 0.09 |
| **FhlA** | 0.05 | 0.05 | 0 |
| **FIS** | 0.14 | 0.08 | 0.05 |
| **FlhD** | 0.25 | 0.25 | 0 |
| **FNR** | 0.5 | 0.1 | 0.1 |
| **FruR** | 0.69 | 0.28 | 0.18 |
| **FucR** | 0.22 | 0.18 | 0.04 |
| **Fur** | 0.83 | 0.15 | 0.14 |
| **GadW** | 0.7 | 0.4 | 0.09 |
| **GadX** | 0.48 | 0.24 | 0.24 |
| **GalR** | 0.22 | 0.22 | 0 |
| **GalS** | 0.47 | 0.36 | 0.11 |
| **GcvA** | 0.6 | 0.26 | 0.17 |
| **GlcC** | 0.75 | 0.7 | 0.06 |
| **GlpR** | 0.35 | 0.13 | 0.1 |
| **GntR** | 0.23 | 0.18 | 0.05 |
| **IclR** | 0.13 | 0.13 | 0 |

| | | | |
|---|---|---|---|
| **IlvY** | 0.51 | 0.51 | 0 |
| **KdpE** | 0.06 | 0.06 | 0 |
| **LexA** | 0.59 | 0.19 | 0.17 |
| **LrhA** | 0.18 | 0.18 | 0 |
| **Lrp** | 0.28 | 0.18 | 0.08 |
| **LysR** | 0.83 | 0.31 | 0.19 |
| **MarA** | 0.81 | 0.81 | 0 |
| **MarR** | 0.41 | 0.41 | 0 |
| **MelR** | 0.19 | 0.19 | 0 |
| **MetJ** | 0.84 | 0.32 | 0.27 |
| **MetR** | 0.04 | 0.01 | 0.02 |
| **MhpR** | 0.32 | 0.25 | 0.08 |
| **Mlc** | 0.67 | 0.4 | 0.17 |
| **ModE** | 0.3 | 0.15 | 0.07 |
| **Nac** | 0.91 | 0.17 | 0.18 |
| **NadR** | 0.22 | 0.12 | 0.09 |
| **NagC** | 0.24 | 0.19 | 0.04 |
| **NarL** | 0.55 | 0.09 | 0.12 |
| **NarP** | 0.15 | 0.15 | 0 |
| **NhaR** | 0.12 | 0.06 | 0.04 |
| **NtrC** | 0.71 | 0.22 | 0.1 |
| **OmpR** | 0.13 | 0.12 | 0.02 |
| **OxyR** | 0.04 | 0.04 | 0 |
| **PdhR** | 0.88 | 0.88 | 0 |
| **PhoB** | 0.83 | 0.2 | 0.22 |
| **PhoP** | 0.25 | 0.13 | 0.06 |
| **PurR** | 0.71 | 0.27 | 0.18 |
| **RcsB** | 0.11 | 0.11 | 0 |
| **RhaR** | 0.23 | 0.23 | 0 |
| **RhaS** | 0.08 | 0.08 | 0 |
| **Rob** | 0.12 | 0.07 | 0.03 |
| **RtcR** | 0.11 | 0.11 | 0 |
| **SlyA** | 0.11 | 0.10 | 0.01 |
| **SoxR** | 0.12 | 0.12 | 0 |
| **SoxS** | 0.11 | 0.07 | 0.02 |
| **TorR** | 0.55 | 0.39 | 0.18 |
| **TreR** | 0.68 | 0.68 | 0 |
| **TrpR** | 0.22 | 0.22 | 0 |
| **TyrR** | 0.06 | 0.03 | 0.01 |
| **UhpA** | 0.13 | 0.13 | 0 |
| **XapR** | 0.2 | 0.2 | 0 |
| **XylR** | 0.56 | 0.56 | 0 |
| **YiaJ** | 0.31 | 0.31 | 0 |
| **Global** | 1 | 0.21 | 0.07 |
