# Supplementary material for: Impact of Transcription Units rearrangement on the evolution of the regulatory network of gamma-proteobacteria
Source: BMC Genomics. 2008 Mar 17;9:128. doi: 10.1186/1471-2164-9-128 (PMC2329645; doi:10.1186/1471-2164-9-128)
Supplement: Additional file 5 — R4: Conservation of regulatory sites and chromosome vicinity of their target genes [file 1471-2164-9-128-S5.pdf]

R4: Conservation of regulatory sites and chromosome vicinity of their target genes

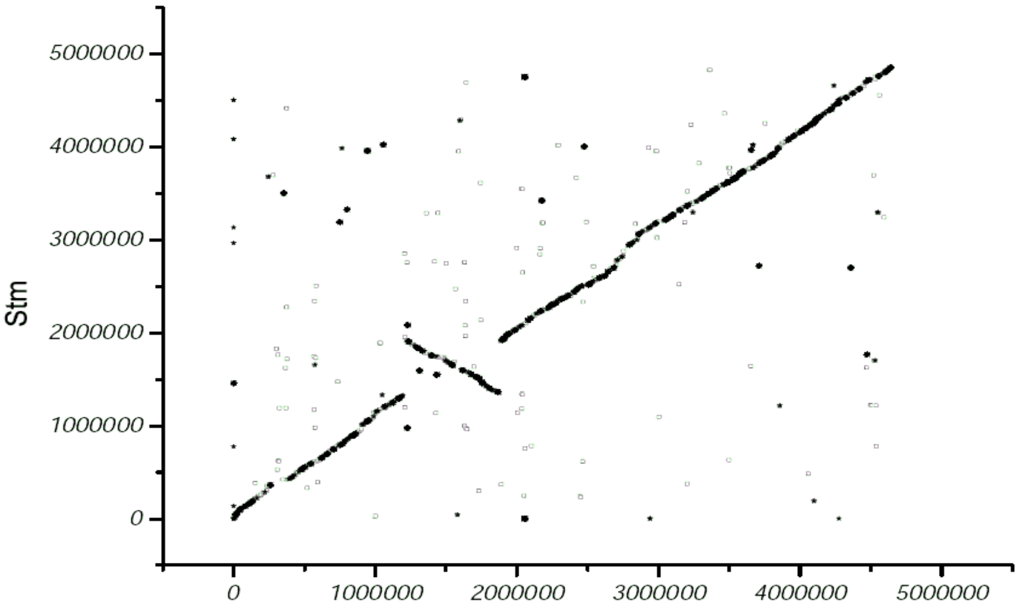

A

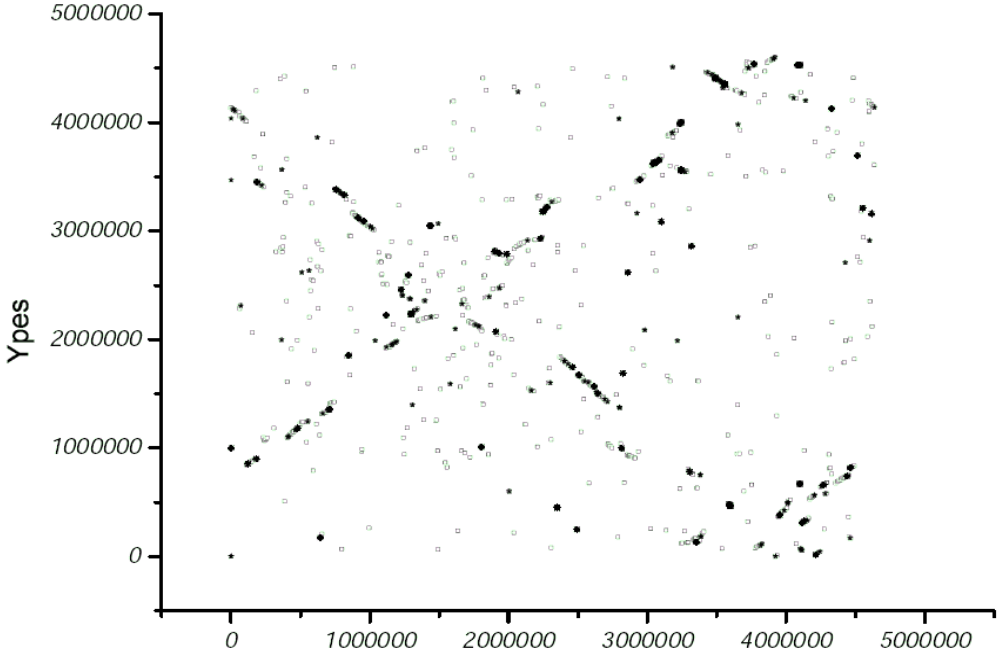

B

Conserved genes and regulatory sites between the Eco and the Stm (A), and the Eco and the Ypes (B) genomes. Pairs of orthologous genes between each pair of genomes have x and y coordinates that correspond to their positions in each genome. Genes that share the same regulation in both genomes are represented as filled squares; genes that belong to the **other regulon** or **lost link** categories are represented as open squares.
