# Supplementary material for: Impact of Transcription Units rearrangement on the evolution of the regulatory network of gamma-proteobacteria
Source: BMC Genomics. 2008 Mar 17;9:128. doi: 10.1186/1471-2164-9-128 (PMC2329645; doi:10.1186/1471-2164-9-128)
Supplement: Additional file 6 — R5: Assessing the quality of reconstructed TRNs using expression data [file 1471-2164-9-128-S6.pdf]

R5: Assessing the quality of reconstructed TRNs using expression data

We used a similar approach to the one described by Babu *et al*. [7]. Briefly, we downloaded *E. coli* gene expression data (22 microarray experiments) from the database University of Oklahoma's E. coli Gene Expression Database (chase.ou.edu/oubcf), and *S. typhimurium* gene expression data (8 microarray experiments) from the Stanford Microarray Database (genome-www5.stanford.edu). Then, we extracted all pairs of co-regulated (or putatively co-regulated) genes from the *E. coli* TRN (retrieved from RegulonDB [8]), and the *E. coli* and *S. typhimurium* TRNs reconstructed using our approach (and stored in Tractor_DB). A pair of co-regulated genes is a pair of genes regulated by the same TF (or exactly the same set of TFs). Finally, we calculated the Pearson Correlation Coefficients of the log-ratios corresponding to all pairs of co-regulated (or putatively co-regulated) genes, and to 1000 randomly chosen gene pairs of each genome. The results are shown in the Figure:

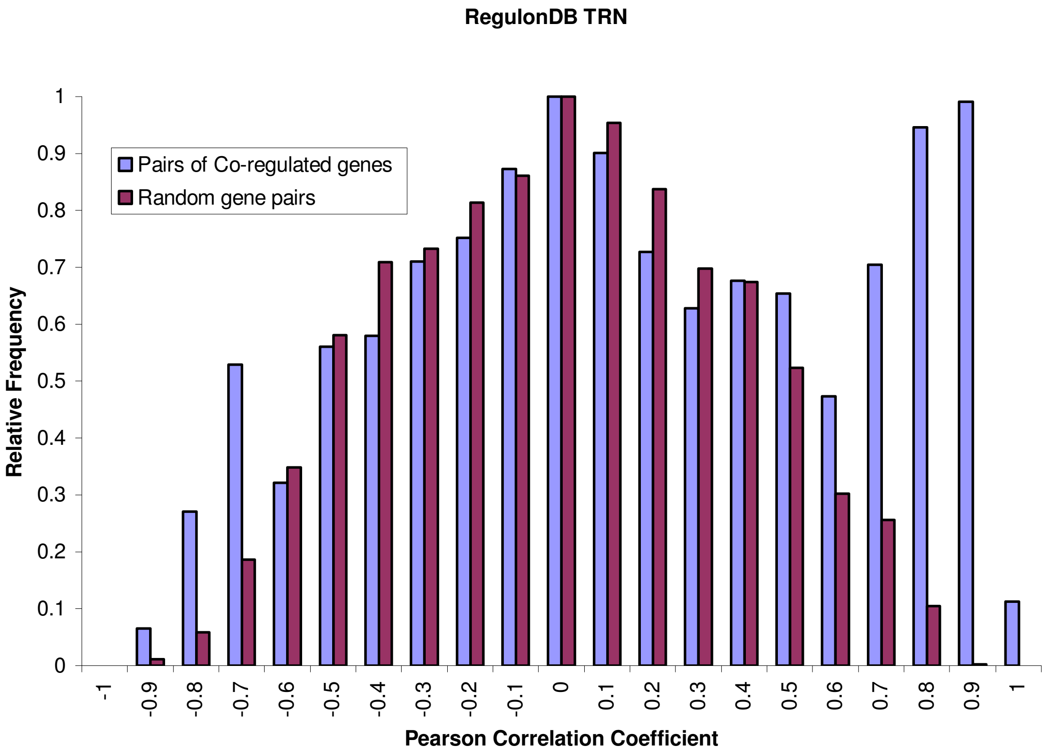

A

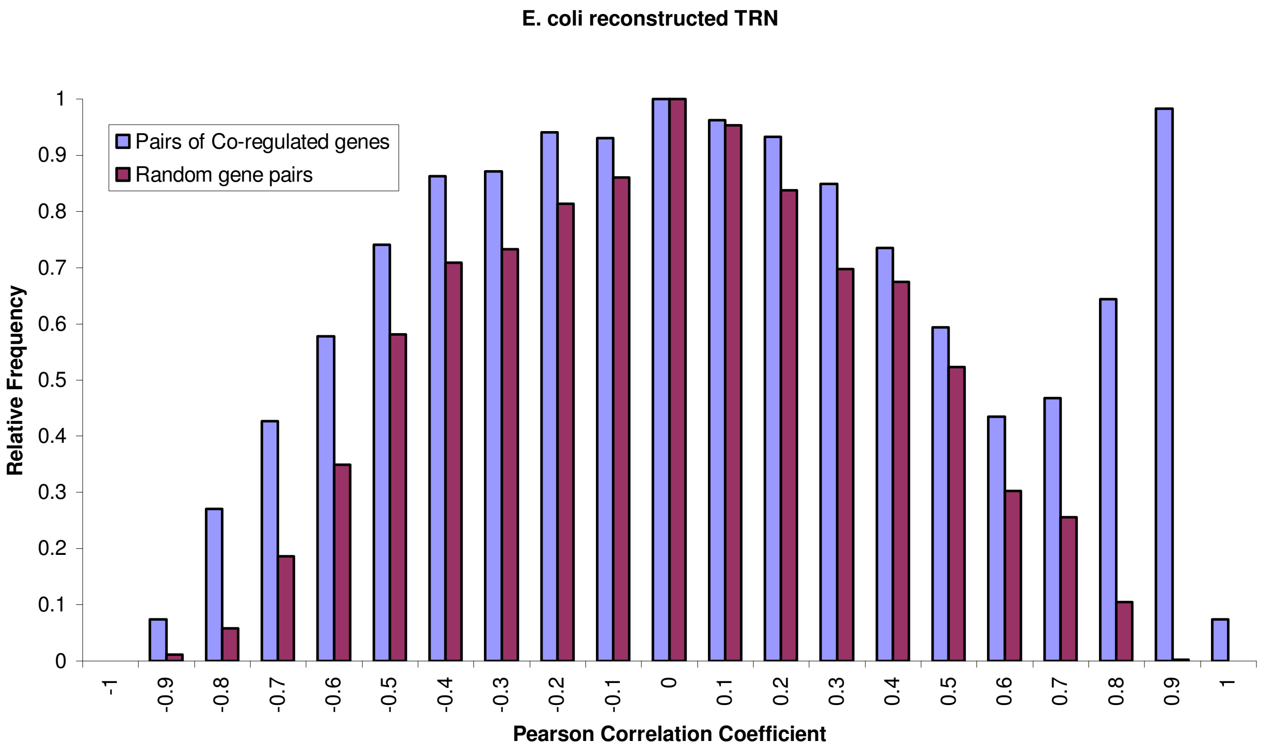
E. coli reconstructed TRN
Pairs of Co-regulated genes
Random gene pairs
Relative Frequency
1
0.9
0.8
0.7
0.6
0.5
0.4
0.3
0.2
0.1
0
-1
-0.9
-0.8
-0.7
-0.6
-0.5
-0.4
-0.3
-0.2
-0.1
0
0.1
0.2
0.3
0.4
0.5
0.6
0.7
0.8
0.9
1
Pearson Correlation Coefficient

B

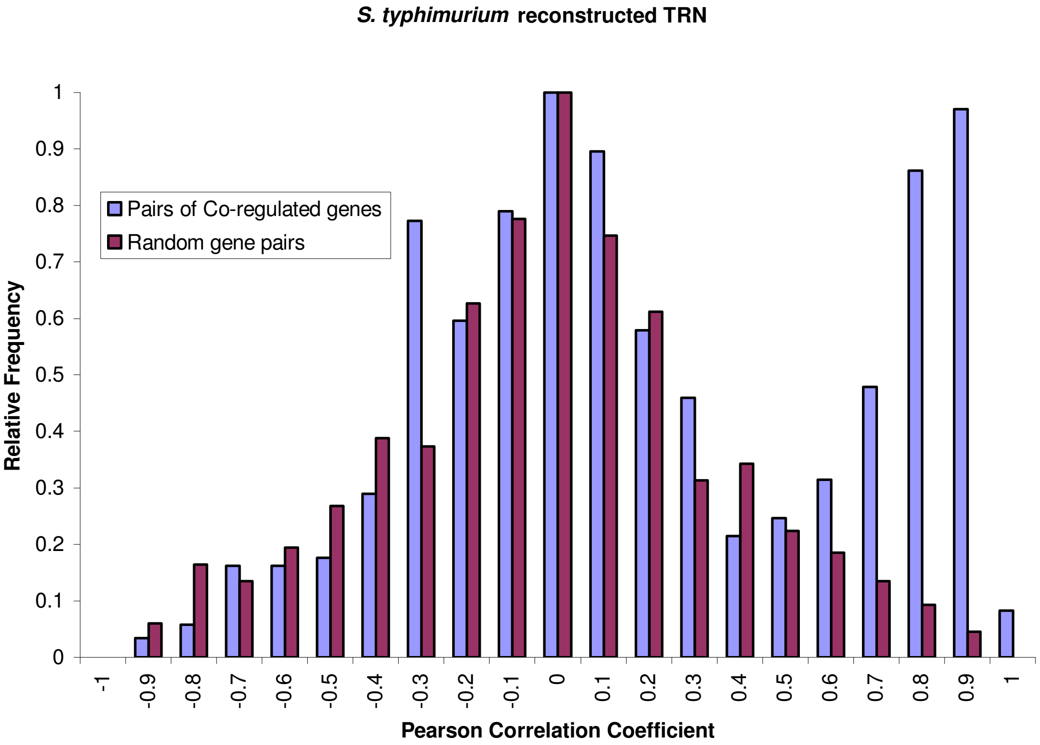
S. typhimurium reconstructed TRN
Pairs of Co-regulated genes
Random gene pairs
Relative Frequency
1
0.9
0.8
0.7
0.6
0.5
0.4
0.3
0.2
0.1
0
-1
-0.9
-0.8
-0.7
-0.6
-0.5
-0.4
-0.3
-0.2
-0.1
0
0.1
0.2
0.3
0.4
0.5
0.6
0.7
0.8
0.9
1
Pearson Correlation Coefficient

C

A: Normalized Pearson Correlation Coefficient distribution for 5138 pairs of experimentally verified co-regulated *E. coli* genes (blue) and 10000 randomly selected gene pairs (red). An important fraction of co-regulated genes possess high correlation coefficients; the peak at 0.8-0.9 does not appear in the normalized distribution of randomly selected gene pairs, indicating that genes under the regulation of the same set of TFs in the *E. coli* TRN tend to show the same expression patterns. **B**: Normalized Pearson Correlation Coefficient distribution for 4089 pairs of computationally predicted co-regulated *E. coli* genes (blue) and 10000 randomly selected gene pairs (red). The shape of the graph is very similar to A (with the same peak at 0.8-0.9), implying that the computationally reconstructed *E. coli* TRN (see text) behaves the same way (when compared to gene expression experiments) than the experimentally verified TRN. **C**: Normalized Pearson Correlation Coefficient distribution for 3577 pairs of computationally predicted co-regulated *S. typhimurium* genes (blue) and 10000 randomly selected gene pairs (red). Again, the shape of the graph (and more precisely the peak at 0.8-0.9) is very similar to A and B. These results strongly support the validity of computationally predicted TF binding sites stored in Tractor_DB.
