# Supplementary material for: Impact of Transcription Units rearrangement on the evolution of the regulatory network of gamma-proteobacteria
Source: BMC Genomics. 2008 Mar 17;9:128. doi: 10.1186/1471-2164-9-128 (PMC2329645; doi:10.1186/1471-2164-9-128)
Supplement: Additional file 7 — R3: SOS values of E. coli regulatory sites against mean scores of their orthologous sites [file 1471-2164-9-128-S7.pdf]

R3: SOS values of *E. coli* regulatory sites against mean scores of their orthologous sites

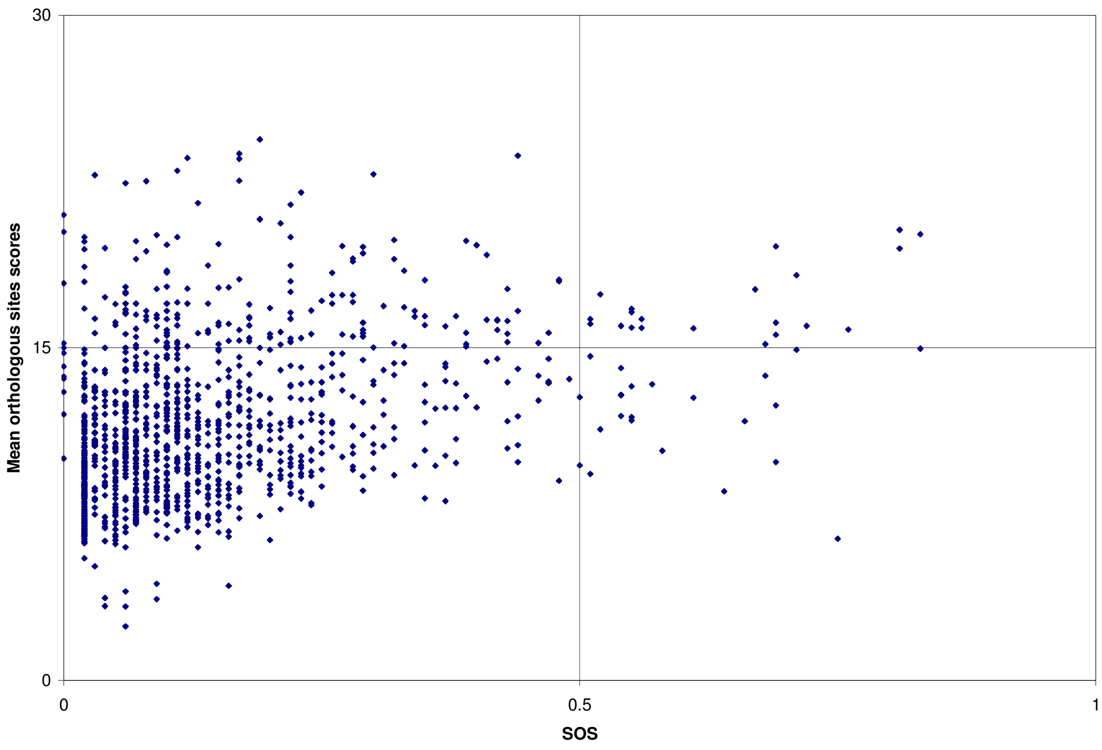

The figure shows the relationship between the SOS values of *E. coli* regulatory sites and the mean scores of their orthologous sites in the other 29 genomes in the study (calculated employing the regulatory models used to identify them). As discussed in the text if the differences of binding affinities between TFs would have had an impact on SOS values, it would be reasonable to expect a large cloud of dots in the low right quadrant. The reason for this would be that weaker statistical models would identify more putative ortholgous regulatory sites (with lower scores due to the low information content of the models) and therefore the corresponding *E. coli* sites would exhibit relatively higher SOS values. The figure clearly shows that the largest cloud of dots is located in the low left quadrant of the graph; this result means that filtering the datasets used to build statistical matrices and selecting the cutoff values for the search of putative orthologous regulatory sites (see Supplementary Material M1 for details) prevented the identification of large number of putative binding sites for TFs with lower binding affinity. This in turn, prevented the appearance of the aforementioned bias in the computation of SOS values.
