# Supplementary material for: Impact of Transcription Units rearrangement on the evolution of the regulatory network of gamma-proteobacteria
Source: BMC Genomics. 2008 Mar 17;9:128. doi: 10.1186/1471-2164-9-128 (PMC2329645; doi:10.1186/1471-2164-9-128)
Supplement: Additional file 8 — R7: Recalculation of the SOS using a different measure of phylogenetic distance [file 1471-2164-9-128-S8.pdf]

R7: Recalculation of the SOS using a different measure of phylogenetic distance

The measure of evolutionary distance that we used to calculate the SOS (namely, the fraction of orthologous genes shared by the genome of a given organism and *E. coli*) may not accurately reflect the phylogenetic distances between these organisms. The identification of pairs of orthologous genes may be influenced by a number of factors (horizontally transferred genetic material, paralogy effects that may give rise to the existence of both inparalogs and outparalogs) that could in principle distort the fractions of orthologous genes calculated.

In order to test the influence of the chosen measure of the evolutionary distance on the distribution of the SOS, we recalculated all the SOS of *E. coli* regulatory links using a different measure of phylogenetic distance. We employed the same measure of distance used by Lozada-Chavez *et al* [19]. In their work, the evolutionary distance from *E. coli* to all organisms was obtained according to the evolutionary branching process reported previously by Brown *et al.* (see full reference below). The evolutionary distance between any two organisms is related to the sum of the distances between each organism and its closest common ancestor. To carry out our calculation of the SOS, we took a unit of phylogenetic distance to be equivalent to 100 aminoacid residue substitutions. Then, we took the inverse of such distances and recalculated the SOS in the manner described in the Methods section. This way, we guarantee that the recalculated SOS are comparable in magnitude to the ones calculated using the fractions of orthologous genes shared by pairs of genomes. The plot of the original SOS *vs* the recalculated SOS is shown in the Figure below. The fairly good correlation between these two measures of conservation of regulatory links suggests that the fraction of shared orthologous genes –although not reproducing exactly the more rigorous measure described above—provides a good approximation of the evolutionary distance between *E. coli* and the other gamma-proteobacteria included in the study. Therefore, a study carried out using a more rigorous measure of phylogenetic distance would most likely arrive to very similar results.

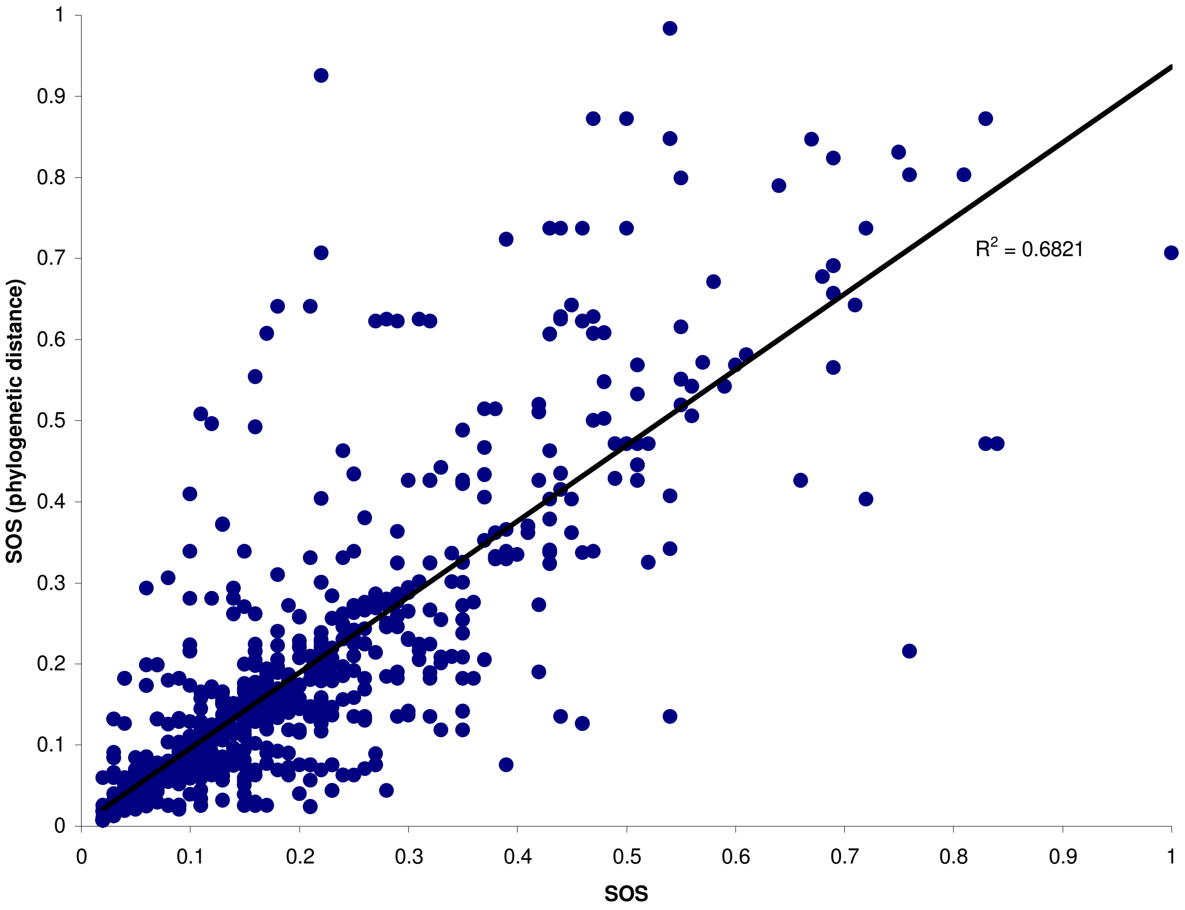

In the figure, each dot corresponds to a single regulatory interaction (or several interactions with the same original SOS and recalculated SOS). The abscissa of each dot corresponds to the original SOS (i.e., the SOS calculated using the fraction of orthologous genes as a measure of phylogenetic distance), and the ordinate corresponds to the recalculated SOS (i.e., the SOS calculated using the measure of phylogenetic distance described above).

Reference

Brown, J.R., Douady, C.J., Italia, M.J., Marshall, W.E. and Stanhope, M.J. (2001) Universal trees based on large combined protein sequence data sets. *Nature Genet.*, **28**, 281–285.
