# Supplementary material for: Impact of Transcription Units rearrangement on the evolution of the regulatory network of gamma-proteobacteria
Source: BMC Genomics. 2008 Mar 17;9:128. doi: 10.1186/1471-2164-9-128 (PMC2329645; doi:10.1186/1471-2164-9-128)
Supplement: Additional file 9 — R2: Calculation of the SOS of a regulatory link [file 1471-2164-9-128-S9.pdf]

R2: Calculation of the SOS of a regulatory link

Fraction of the genes of each organism with orthologs in *E. coli*. The table shows only organisms where we detected orthologs of FruR and the genes of the *fruB-fruK-fruA* TU.

Erwinia carotovora atroseptica SCRI1043	0.93
Shigella flexneri 2a 2457T	0.78
Shigella flexneri 2a	0.77
Haemophilus influenzae	0.71
Escherichia coli O157H7 EDL933	0.70
Escherichia coli CFT073	0.67
Salmonella typhimurium LT2	0.70
Photobacterium profundum SS9	0.68
Salmonella typhi	0.68
Vibrio cholerae	0.62
Pseudomonas syringae	0.61
Vibrio parahaemolyticus	0.60
Vibrio vulnificus CMCP6	0.60
Yersinia pestis KIM	0.56
Yersinia pseudotuberculosis IP32953	0.56
Photorhabdus luminescens	0.42
Yersinia pestis biovar Mediaevails	0.32
Pseudomonas putida	0.31
Pseudomonas aeruginosa	0.31

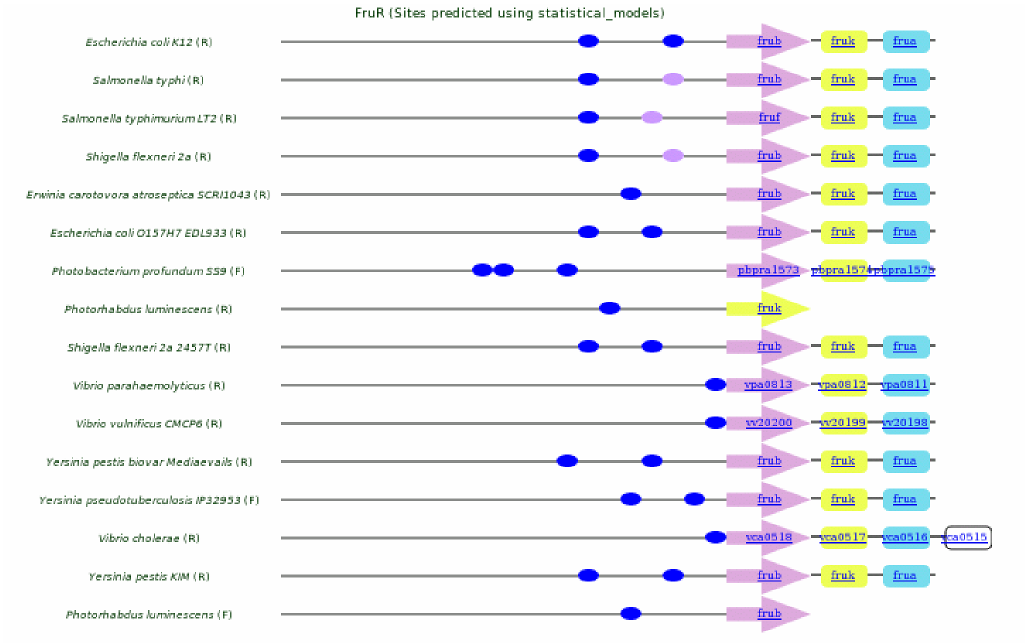

SOS numerator = (1 – 0.68) + (1 – 0.7) + (1 – 0.77) + (1 – 0.93) + (1 – 0.7) + (1 – 0.68) + (1 – 0.42) + (1 – 0.78) + (1 – 0.6) + (1 – 0.6) + (1 – 0.32) + (1 – 0.56) + (1 – 0.62) + (1 – 0.56) = 5.08

SOS denominator = (1 – 0.31) + (1 – 0.77) + (1 – 0.61) + (1 – 0.67) + (1 – 0.7) + (1 – 0.62) + (1 – 0.56) + (1 – 0.71) + (1 – 0.6) + (1 – 0.42) + (1 – 0.68) + (1 – 0.6) + (1 – 0.93) + (1 – 0.68) + (1 – 0.56) + (1 – 0.7) + (1 – 0.78) + (1 – 0.32) + (1 – 0.31) = 7.47

SOS = 0.68
